# Computational pipeline predicting cell death suppressors as targets for cancer therapy

**DOI:** 10.1016/j.isci.2024.110859

**Published:** 2024-08-30

**Authors:** Yaron Vinik, Avi Maimon, Harsha Raj, Vinay Dubey, Felix Geist, Dirk Wienke, Sima Lev

**Affiliations:** 1 Molecular Cell Biology Department, Weizmann Institute of Science, Rehovot 76100, Israel; 2 The Healthcare Business of Merck KGaA, Darmstadt, Germany

**Keywords:** Cell biology, Bioinformatics, Cancer, Transcriptomics

## Abstract

Identification of promising targets for cancer therapy is a global effort in precision medicine. Here, we describe a computational pipeline integrating transcriptomic and vulnerability responses to cell-death inducing drugs, to predict cell-death suppressors as candidate targets for cancer therapy. The prediction is based on two modules; the transcriptomic similarity module to identify genes whose targeting results in similar transcriptomic responses of the death-inducing drugs, and the correlation module to identify candidate genes whose expression correlates to the vulnerability of cancer cells to the same death-inducers. The combined predictors of these two modules were integrated into a single metric. As a proof-of-concept, we selected ferroptosis inducers as death-inducing drugs in triple negative breast cancer. The pipeline reliably predicted candidate genes as ferroptosis suppressors, as validated by computational methods and cellular assays. The described pipeline might be used to identify repressors of various cell-death pathways as potential therapeutic targets for different cancer types.

## Highlights

• TNBC are highly susceptible to ferroptosis, especially the mesenchymal subtype
• A computational pipeline for the detection of targets for cancer therapy is introduced
• The pipeline is based on transcriptomic and vulnerability responses to drugs
• The pipeline can be generalized to other cell-death pathways and to various cancers

## Graphical abstract

**Figure:**
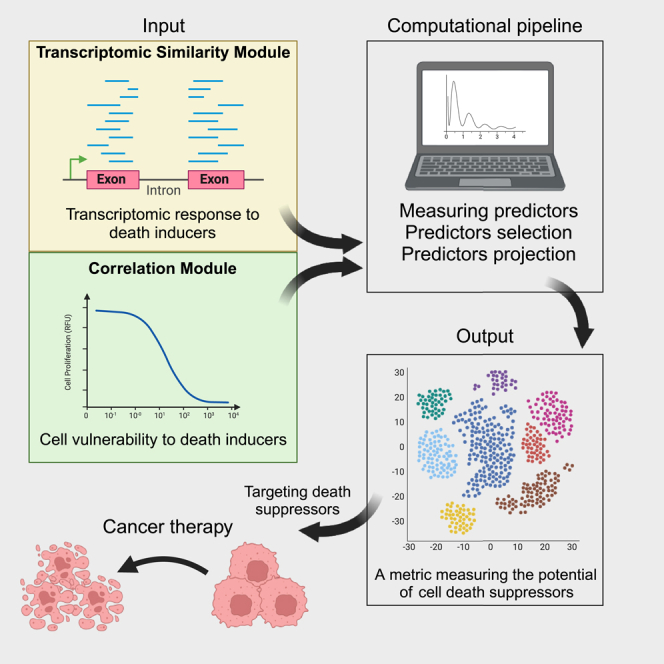

## Introduction

Triple negative breast cancer (TNBC) is an aggressive disease characterized by high metastasis and poor prognosis.^1^ The disease is defined by the lack of estrogen and progesterone receptors and HER2 amplification, and currently, it has no effective, clinically approved therapeutic targets.^2^ Although the efficacies of various targeted therapies have been tested in TNBC pre-clinical models and many of them initially eliminated tumor cells through apoptosis, frequently, the tumor cells develop drug resistance and relapse. In recent years, targeting of non-apoptotic cell-death pathways, such as ferroptosis, has been considered as promising strategy for cancer therapy.^3^ We previously showed that TNBCs are particularly vulnerable to ferroptosis,^4^ implying that this death module can be exploited for TNBC therapy. However, canonical ferroptosis inducers, such as erastin, which inhibits the System Xc^-^ of cystine/glutamate antiporter, and RSL3, which inhibits glutathione peroxidase 4 (GPX4), the major cellular protector of lipid peroxidation, have little efficacy *in vivo* and, therefore, limited clinical implications.^5^ These limitations boosted robust efforts to identify ferroptosis repressors, several have been identified through genetic screens, such as GCH1,^6^ FSP1,^7^ DHODH^8^ and MBOAT1/2.^9^ Additional genes are continuously discovered and might be potent, targetable suppressors with high anti-cancer efficacy.

Several computational methods have been recently developed to identify therapeutic targets utilizing multiomics profiling and machine learning.^10^ These methods can predict the outcome of gene targeting,^11^^,^^12^ employing several publicly available transcriptomic resources such as the connectivity map (CMAP), which includes thousands of perturbational datasets. The CMAP can predict the phenotypic outcome of gene targeting based on similarities between the transcriptomic responses induced by numerous pharmacological or genetic perturbations. This resource has been used for gene targets discovery,^13^ for drug repurposing,^14^ and for predicting highly potent drug combinations.^15^ Other approaches integrate gene expression profiles and susceptibility to various drugs to predict drug targets.^16^ This approach is provided, for example, by the Cancer Dependency Map (DepMap; Broad Institute) portal, which includes large datasets of gene expression, gene essentiality, and drug sensitivity across multiple cancer cell lines.^17^ Combining the transcriptomic and the vulnerability data of cancer cell lines with the data derived from patients with cancer has been previously used to build prediction models connecting gene expression to drug sensitivity.^18^ Since both the transcriptomic and vulnerability data rely on large-scale screens, which might yield false positives, their integration may increase the robustness and confidence of the predicted targets.

Here we employ a computation approach, integrating the transcriptomic and the vulnerability responses of TNBC cells to ferroptosis inducers (FINs), to eventually predict ferroptosis repressors as promising candidate genes for cancer therapy. Practically, we measured the vulnerability of multiple TNBC cell lines to FINs and concomitantly profiled the FINs transcriptomic response in the same TNBC cell lines. This experimental data was combined with public resources (CMAP, DepMAP) to assess the potential of a given gene to suppress ferroptosis based on two modules: a transcriptomic similarity module and a correlation module. The output of the pipeline is a ranked list of genes, which can predict ferroptotic cell death in response to their targeting, at least in TNBC. The prediction is based on the proximity of the candidate gene, on a dimension reduction plane, to other key players of ferroptosis, such as GPX4 and GCH1. The power and reliability of the pipeline were validated experimentally by selecting representative genes from the ranked list and demonstrating their ferroptosis suppressing capacity in TNBC. We propose that the integrated drug discovery pipeline can be used as a resource for targeting different cell death pathways in various cancer lineages.

## Results

### Pipeline design to identify ferroptosis suppressors in triple negative breast cancer

**Figure 1:**
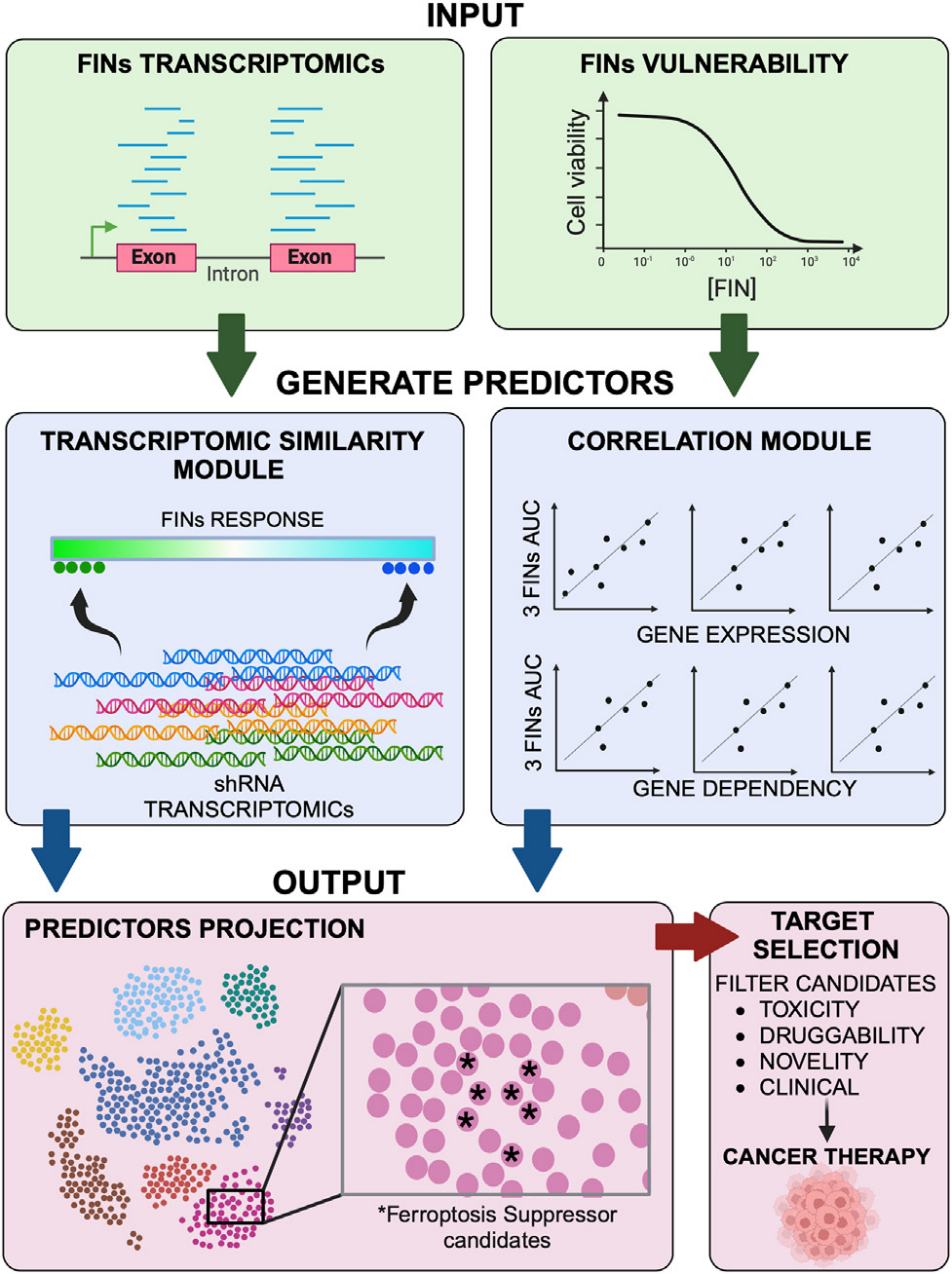
Pipeline workflow The pipeline flow chart. The pipeline was generated to predict candidate genes as ferroptosis suppressors using transcriptomic similarity and correlations modules.

Considering the intrinsic vulnerability of TNBC to ferroptosis,^4^^,^^19^ we constructed a computational pipeline that identified potential suppressors of ferroptosis. We assume that the inhibition of these candidate genes should induce ferroptosis, at least in TNBC. We also assume that some of these targets might be used for cancer therapy, depending on critical considerations (toxicity, druggability, and so forth). The pipeline relies on a set of predictors calculated for each gene (Figure 1). These predictors quantify (i) the similarity between the transcriptomic response induced by targeting the candidate genes to the response induced by the FINs, and (ii) the correlation between the transcription levels of the candidate genes to the vulnerability - of the TNBC cell lines to the FINs. The set of predictors was then integrated into a single score that estimates the potential of each gene targeting to induce ferroptosis. Further computational and experimental validations highlighted the predictive power of the pipeline. The pipeline flow chart is depicted in Figure 1.

### Correlations between transcriptomic response and ferroptosis susceptibility

According to the pipeline design, we first measured the correlations between gene expression or gene essentiality to ferroptosis vulnerability, which was quantified by the area under the dose-response curve (AUCs) in TNBC cell lines. High correlations between the level of gene expression and the susceptibility to FINs as measured by the AUCs suggest that a decrease in the expression of these genes might sensitize cells to FINs, thus highlighting possible ferroptosis suppressors. For gene essentiality, we used the gene dependency scores (provided by the Achilles dataset, Broad Institute), which are given on a negative scale, with lower values indicating higher essentiality. Once again, a high correlation of gene dependency score to FINs AUCs might highlight potential repressors.

**Figure 2:**
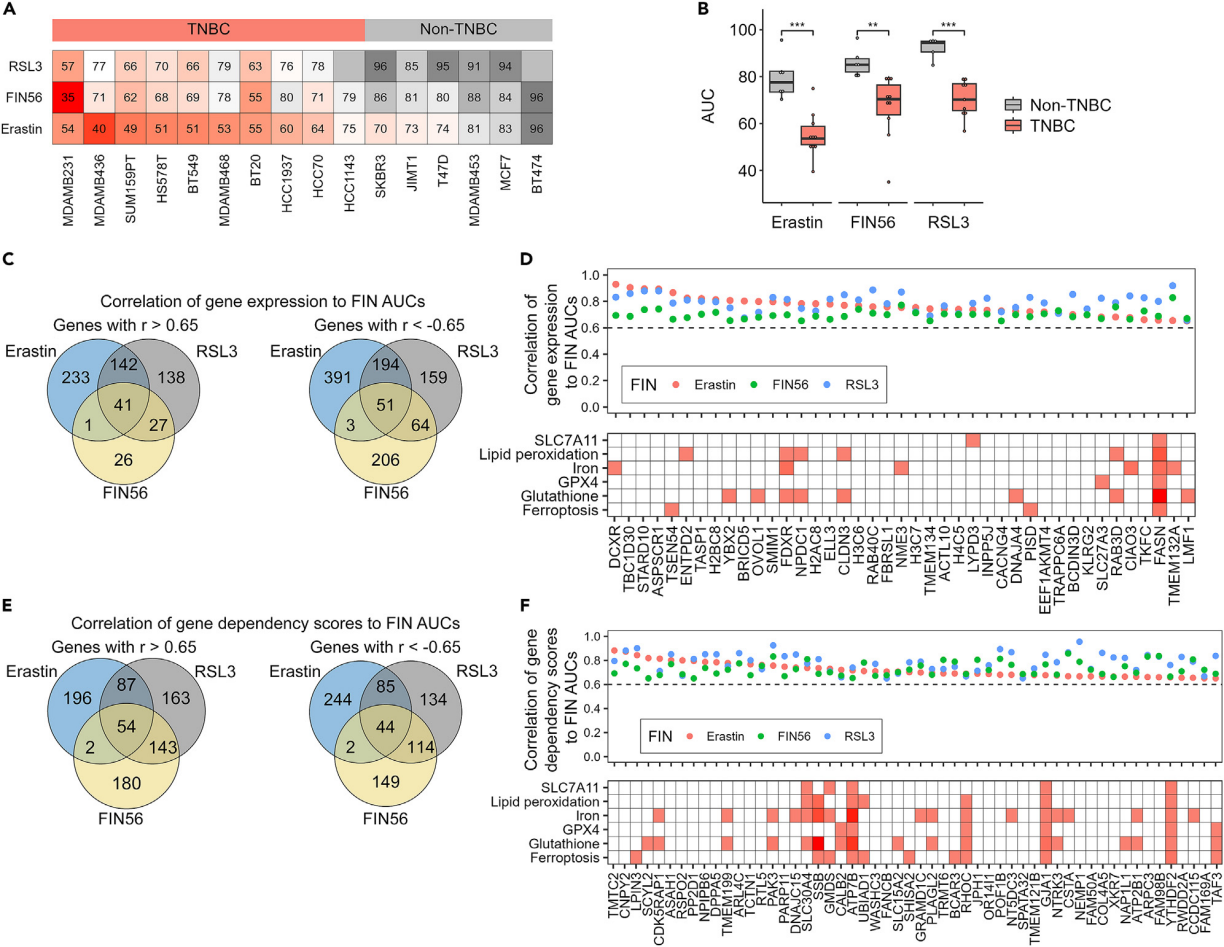
Assigning the correlation predictors for each gene (A and B) Area under the curves (AUCs) were quantified from 16 dose-response curves obtained in the indicated breast cancer cell lines in response to erastin, RSL3, or FIN56 treatment. High AUCs represent a low vulnerability to the FINs. T-test (in B) was used to evaluate *p*-values (∗∗*p*-value<0.01, ∗∗∗*p*-value<0.001). The AUCs are averages of 3–4 repeats for each cell line and inducers. (C–F) Pearson’s correlations were calculated between the expression level of genes (CCLE data, panels C and D) or the essentiality of genes (Achilles data, panels E and F) and the FIN AUCs. The Venn diagrams (C and E) show the number of genes with correlation above 0.65 or below −0.65. The genes with correlation above 0.65 for all 3 FINs are shown in (D) (gene expression vs. AUCs) or (F) (gene dependency vs. AUCs). In both panels, the top plot shows the correlations of the expression (D) or the dependency (F) to the 3 FINs. The bottom plot shows the results of a PubMed search for each gene with 6 ferroptosis related terms. A red box indicates citation(s) of the gene in the related term, while the color hue corresponds to the citation number.

We previously showed that TNBCs are more susceptible to FINs (erastin and FIN56) compared to cell lines derived from other breast cancer subtypes.^4^ To expand these findings, we also assessed the susceptibility of TNBC and non-TNBC cell lines to RSL3, thereby analyzing the effects of the three canonical FINs of the major FIN classes (class 1- erastin, class 2-RSL3, and class 3-FIN56)^20^ using dose-response curves (Figure S1A). We quantified the area under the curves (AUCs), as a measure of ferroptosis susceptibility (Figure 2A), and observed, as expected, that TNBC are more susceptible to ferroptosis (Figure 2B).

Next, we correlated the AUCs of the 16 BC cell lines to gene expression (CCLE data) and gene dependency scores (Achilles data, Broad Institute). We found 41 genes with a high correlation (r > 0.65) between gene expression levels and the AUCs of the 3 FINs (Figures 2C and 2D). This analysis highlighted DCXR (dicarbonyl and L-xylulose reductase) as the highest correlative gene to erastin AUC, and ASPSCR1 (Tether containing UBX domain for GLUT4) as the highest correlative gene to all three FINs. Neither of them have been reported, thus far, as ferroptosis regulators (Figure 2D). Gene ontology analysis of these 41 genes revealed a significant enrichment of nucleosome-related genes (Figure S1B), which might be relevant for the ferroptosis related effect of HDAC inhibitors.^21^^,^^22^

Similarly, we found 54 genes with a high correlation of gene dependency to the AUCs (Figures 2E and 2F). In total, for each gene we acquired 6 correlation scores (correlation of the gene expression or the gene dependency to the AUC of the three FINs: erastin, RSL3 and FIN56) (Table S1). These scores will be henceforth termed the “correlation predictors”.

### Transcriptomic response to ferroptosis inducers in triple negative breast cancer

The assigned 6 predictors described above rely on the correlations to FIN vulnerabilities, suggesting that genes with high predictor values would be associated with increased ferroptosis vulnerability. However, since correlations might not imply causation, we assumed that introducing an additional set of predictors could increase the confidence in the target selection process. We, therefore, introduced the transcriptomic similarity predictors, which reflect the similarity between the transcriptomic response to FINs treatment and the transcriptomic response of gene targeting.

**Figure 3:**
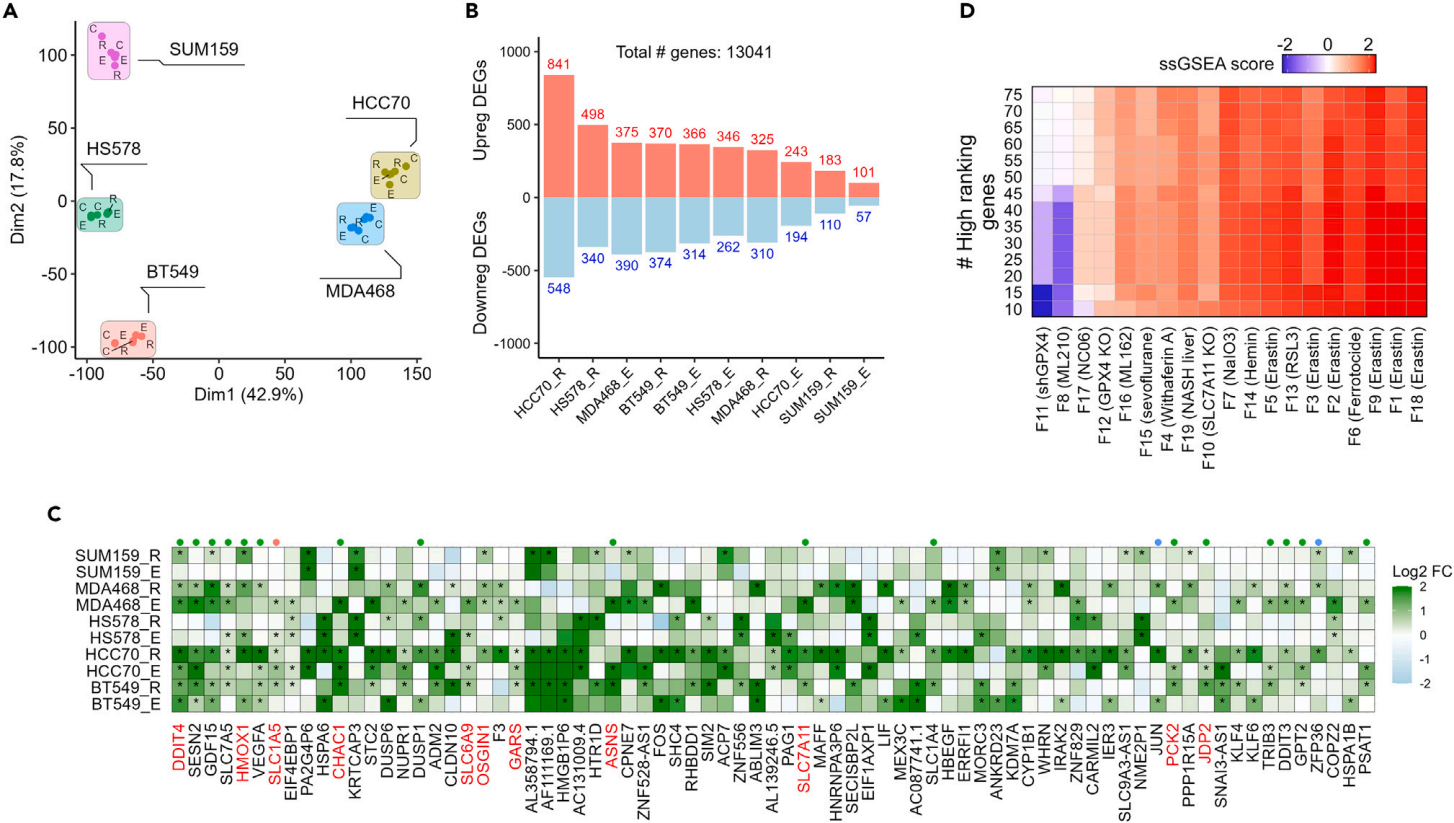
Transcriptomic analysis of FINs response in TNBC (A–C) RNAseq was performed for 5 TNBC cell lines, treated either with erastin (marked with “E”), RSL3 (“R”), or DMSO as control (“C”). Experiments were done in duplicates. (A) PCA analysis of the RNAseq data. (B) The number of significant (*p*-value <0.05) differentially expressed genes was determined for each treatment vs. control (“_E” denotes erastin vs. DMSO, “_R” denotes RSL3 vs. DMSO). (C) Heatmap showing the 75 genes with the highest average fold change expression between the FINs and DMSO. The points above the heatmap indicate genes that appear in the FerrDB and are known as ferroptosis markers (green), drivers (red), or suppressors (blue). The genes in red are ferroptosis-to-apoptosis biomarkers, as previously described.^23^ (D) The enrichment of the top 10–75 genes from the heatmap in (C) was assessed in 19 public FIN datasets (Table S3). The enrichment was performed using ssGSEA.

To that end, we profiled the transcriptomic response of FINs, by treating 5 TNBC cell lines of different molecular subtypes, including two basal-like (BL) (MDA-MB-468, HCC70), and three mesenchymal-like (M) (Hs578, SUM-159, BT549), with either erastin or RSL3. We then performed RNAseq to determine the transcriptomic effect of those FINs in the TNBC cell lines. Principal component analysis (PCA) showed that the diversity in the transcriptomic response is mainly associated with differences between the cell lines, rather than the FIN identity (Figure 3A). This was further corroborated by the relatively small number of significant differentially expressed genes (DEGs) between erastin- or RSL3-treated cells vs. DMSO-treated cells (Figure 3B). Among the 10 RNAseq datasets (5 cell lines X 2 FINs), 126 genes were significantly up-regulated in 3 or more datasets (Figures S2A and S2B; Table S2), including 14 genes, which we recently identified as ferroptosis vs. apoptosis biomarkers.^23^ Analysis of the transcriptomic responses to FINs did not reveal specific expression patterns associated with either the subtype of the TNBC cell lines (BL vs. M) or the identity of the FINs (erastin vs. RSL3) (Figure S2A). However, gene set enrichment analysis (GSEA) showed enrichment of genes related to amino acid deprivation,^24^ and response to oxidized phospholipids, among other pathways that could be relevant to ferroptosis (Figure S2C). All genes were than ranked by their average fold change in expression in response to FINs versus DMSO. Figures 3C and S2D depict the 75 genes with the highest (Figure 3C) and lowest (Figure S2D) average fold change, representing the most up- and down-regulated genes in response to the applied FINs. Among the 75 up-regulated genes, 20 genes have already been identified as ferroptosis associated genes (FerrDB database,^25^), including genes with anti-ferroptotic effects, such as PCK2^26^ or SLC1A5,^27^ among others.

To further characterize this FINs-induced gene set in TNBC and to assess whether it represents a general transcriptomic landscape of ferroptotic response, we collected transcriptomic profiles of 19 publicly available ferroptosis responses to different chemical and genetic perturbations (Table S3) from GEO. Scoring the enrichment of the top-ranking genes (10–75) in the heatmap (Figure 3C) by ssGSEA revealed high enrichment in most of the 19 GEO profiles (Figure 3D), highlighting the importance of these genes in the general transcriptomic response to FINs.

### Connectivity map analysis and extraction of transcription similarity scores

The second applied approach for ferroptosis target discovery was associated with transcriptomic similarity. In this approach, we assessed the similarities between the transcriptomic response of FINs obtained in TNBC cell lines (Figure 3C), to that of other chemical or genetic perturbations obtained from the Connectivity Map (CMAP). The CMAP database includes ∼475,000 transcriptomic profiles in 4–9 cancer cell lines in response to ∼19,800 small molecules and ∼7,500 genetic perturbations.^28^ Usually, the CMAP signatures are compared to a gene set of interest. In our case, we delved into the CMAP signatures database using a gene set composed of the 20 genes with the highest average fold change in their expression in response to the FINs in the 10 datasets as shown in Figure 3C. Indeed, we observed high scores for signatures of canonical FINs such as erastin and sorafenib of shRNA of GPX4. (Figure S2E).

**Figure 4:**
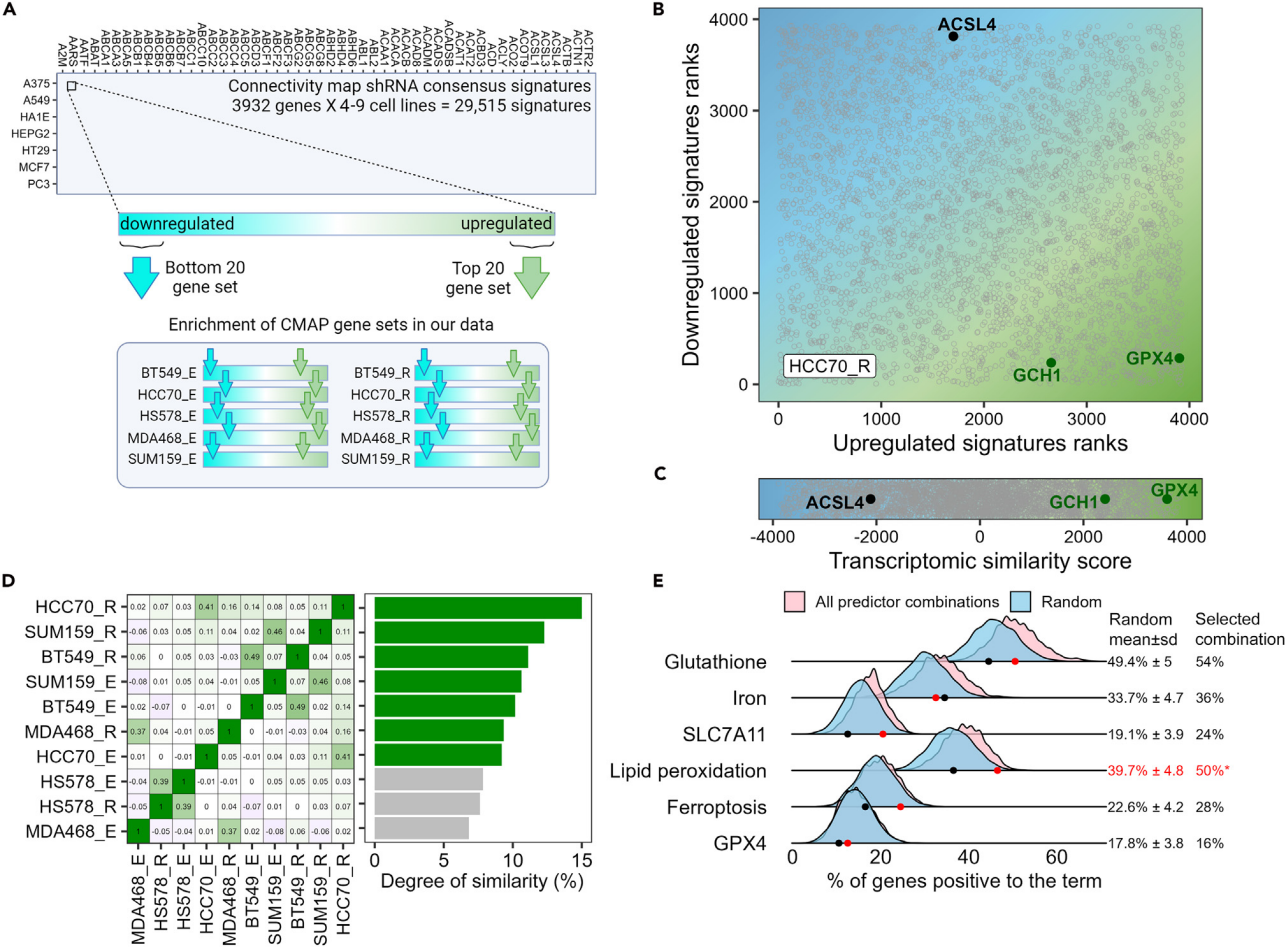
Assigning the transcriptomic similarity scores for each gene (A) The shRNA consensus transcriptomic profiles were downloaded from the CMAP database. Up- and down-regulated gene sets were extracted, and their enrichment in the 10 RNAseq datasets (5 TNBC cells x 2 FINs) was measured using the CAMERA method. (B) Plot showing the ranked transcriptomic similarity scores calculated for one representative of the 10 datasets (HCC70 treated with RSL3). In this plot, ∼3900 points are shown, each representing an shRNA from the CMAP database. The x- and y axis are the ranked similarity scores calculated using the upregulated and downregulated gene sets. A potential ferroptosis suppressor is predicted to induce a transcriptomic profile in which the top and the bottom 20 genes are ranked high and low, respectively, in the 10 RNAseq datasets, and therefore should be positioned in the bottom-right corner of the plot (green area). (C) The final similarity score for each gene in each treatment was calculated as the upregulated rank minus the downregulated rank. The plot shows these scores for all genes in HCC70 treated with RSL3, as a representative. (D) Predictor selection method. The matrix (left) shows the pairwise Pearson’s correlations between the 10 transcriptomic profiles of FINs in TNBC (shown in Figure 3C). Correlations were calculated using the fold change values of all genes in each profile. Based on these correlations, a metric was evaluated describing how each transcriptomic profile is similar to the consensus of all 10 profiles (right). To calculate this metric, the correlations in each row of the matrix were summed up, excluding the negative correlations and the correlations in the diagonal (which equal 1). These summations were then normalized on a scale of 0–100 and are shown in the plot to the right. The green bars represent the predictors with the highest similarity to the consensus of all profiles, while the gray bars are the predictors with the lowest such similarity, which were filtered out in the next steps. (E) Validation of predictor selection by text-mining. Sets of genes were delved in PubMed (using the GeneShot tool), to measure the percentage of genes in each set which is cited with the terms shown in the y axis. The pink density plots represent 1023 combinations of predictors, in each the top 50 genes with the highest values of selected predictors were analyzed. In blue, 2000 sets of randomly selected genes were similarly analyzed. The number on the left indicates the mean ± standard deviation of the random sets. The red dot, and the numbers under “selected combination,” represent the actual predictor combination selected for this study (the ∗ indicates *p*-value <0.05, comparing the selected combination to the random distribution). The black dot represents the predictor combination which includes all the predictors generated.

Nevertheless, previous reports highlighted the variable transcriptomic response of a given perturbation across different cell lines.^29^ Therefore, instead of comparing the combined gene set of the 20 genes derived from all the 10 datasets described in Figure 3C, we used a cell-specific approach and examined the similarities between the CMAP signatures (29,515 signatures = 3932 knockdown genes X 4–9 cell lines) in each individual transcriptomic response of the 10 FINs datasets (Figure 4A).

We used the 29,515 shRNA consensus signatures from the CMAP and extracted from each signature the top 20 and bottom 20 genes, which represent the most upregulated and downregulated genes of each signature. This yielded 59,030 gene sets in total. Next, we used the CAMERA method^30^ to quantify the enrichment of those gene sets in the 10 transcriptomic datasets of FINs (Figure 4A; Table S4). These scores, henceforth termed the “Transcriptomic Similarity Scores,” represent the similarity between the transcriptomic profiles of ferroptosis induction and the transcriptomic profile of the 3932 knocked-down (KD) genes.

For each KD gene (shRNA), the CMAP database contains 4–9 transcriptomic profiles, one for each cell line in the CMAP project. The 4–9 transcriptomic similarity scores of each shRNA were aggregated into one by selecting the maximum value (Figure S3A). We then ranked the shRNA based on their similarity scores (both for the upregulated and the downregulated gene sets) (Figure S3B). The calculated ranked similarity scores of the 3932 shRNAs to the transcriptomic response of HCC70 cells treated with RSL3 are shown in Figure 4B as an example. The x- and y axis of the plot are the ranked similarity scores calculated using the upregulated (x axis) and downregulated (y axis) gene sets. Promising ferroptosis suppressor candidates should, therefore, be ranked high among the upregulated gene sets similarity scores (Figure S3B, left panel), and low among the downregulated gene sets similarity scores (Figure S3B, right panel). Indeed, GPX4 and GCH1, two canonical ferroptosis repressors, fulfill these criteria, as seen in Figure 4B (both appear at the lower right corner of the plot).

Next, we combine the upregulated and downregulated ranks into a single metric for each shRNA by subtracting the downregulated rank from the upregulated rank. The obtained final scores ranged from ∼4000 (best potential ferroptosis suppressors) to ∼ -4000 (best potential ferroptosis inducers) for each shRNA (Figure 4C). These 10 scores per shRNA (one for each transcriptomic dataset of FINs in TNBC) reflect the similarities between the transcriptomic response of each shRNA from the CMAP to the transcriptomic response of the FINs and were considered to be the 10 transcriptomic similarity predictors. For most predictors, the values are associated with known ferroptosis suppressors. For example, the 40 genes from the FerrDB database of ferroptosis suppressors, which were also analyzed in the CMAP database, were indeed highly enriched compared to other genes (Figure S3C).

To further optimize the pipeline, we introduced a predictor selection step. This step was applied due to the variability in the transcriptomic responses of FINs in TNBC (Figure S2A), which was associated with a high variance between the 10 transcriptomic similarity predictors for each shRNA. To reduce the predictors' variance, we calculated the consensus level between the 10 transcriptomic profiles induced by the FINs in TNBC (Figure 4D, see STAR Methods for details) and then removed the 3 predictors with the lowest similarity to the consensus of all the 10 profiles. This predictor selection step resulted in 7 optimized transcriptomic similarity predictors, which were further combined to the 6 correlation predictors calculated earlier (Figure 2).

To demonstrate the advantage of the predictor selection step, which reduced the number of the transcriptomic similarity predictors from 10 to 7, we used a text-mining analysis. In brief, the 6 correlation predictors were combined with any possible combination of the 10 original transcriptomic similarity predictors (1023 predictor combinations in total), and the top 50 genes for each combination with the highest predictor values were selected. We then calculated the percentage of genes, among these 50 genes, cited with ferroptosis related terms in PubMed (Figure 4E). To evaluate the statistical significance of this analysis, we similarly analyzed randomly selected sets of genes. This analysis indicated that combining the 6 correlation predictors with the 7 selected transcriptomic similarity predictors yields a list of genes with high enrichment for genes cited with those terms, in particular with the term “lipid peroxidation,” compared to most predictor combinations.

### Integrating the transcriptomic similarity scores with the correlation scores

**Figure 5:**
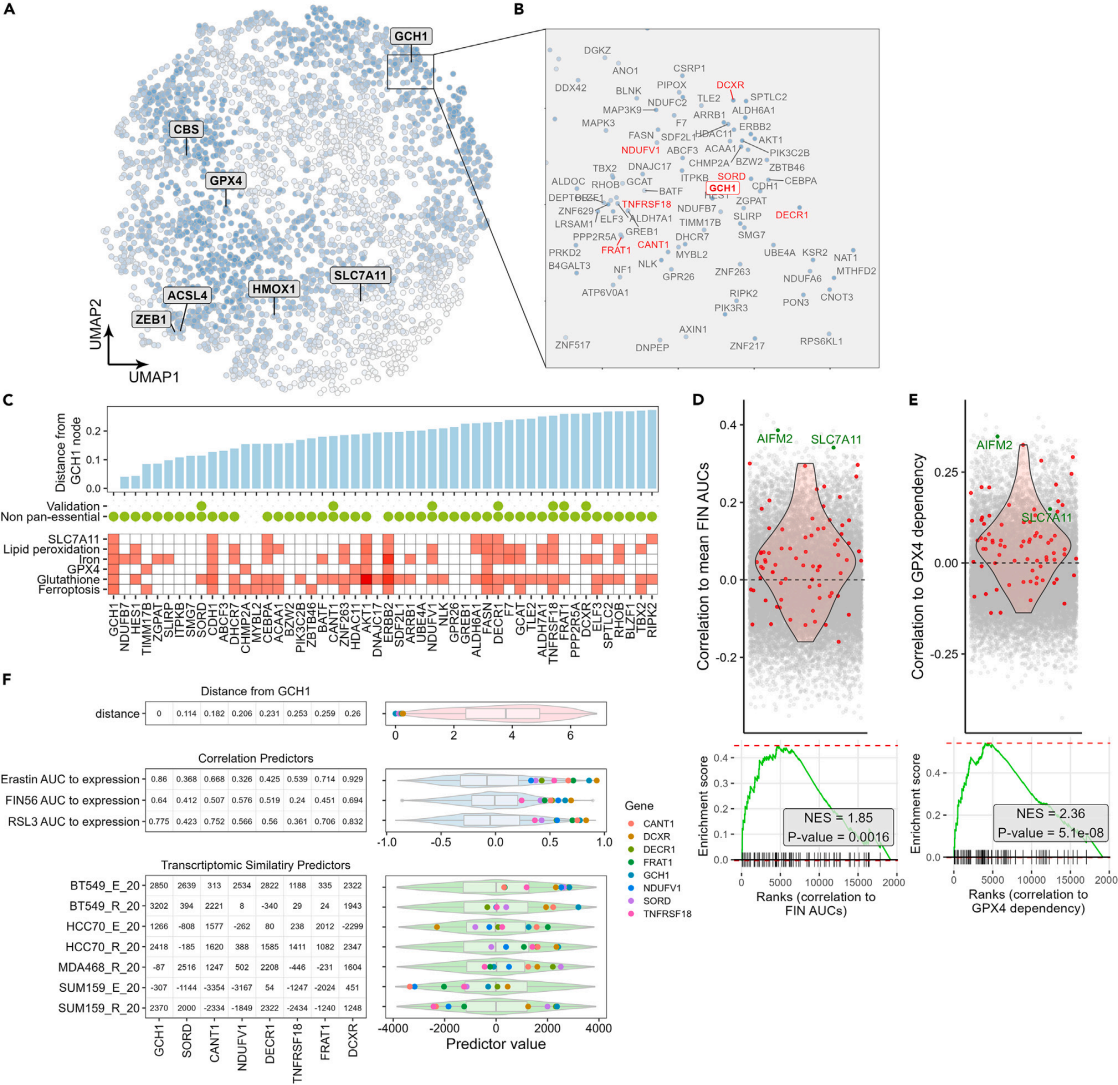
Integrating the transcriptomic similarity and correlation scores to predict potential ferroptosis suppressors (A) UMAP projection of 13 scores (7 transcriptomic similarity and 6 correlations score) per gene. A selection of known ferroptosis regulators are labeled. The blue scale of the dots (genes) represents an averaging of all the predictor values (see STAR Methods section). (B) Zoom-in on the GCH1 node neighborhood in the UMAP. (C) Genes from the GCH1 neighborhood (shown in B), organized by their Euclidean distance from the GCH1 node (top panel). The bottom plot is a PubMed search of each gene with 6 ferroptosis related terms. Red boxes indicate citation(s) with the specified term, while the color hue corresponds to the number of citations. Genes that are not essential pan-cancer, as well as those selected for experimental validation, are marked by green dots. (D and E) Correlations of gene expression levels (CCLE pan-cancer cell line data) to the dependency score of GPX4 (taken from the Achilles dataset) (in E) or to the AUCs of 4 FINs taken from the CTRP dataset (in D, shown is the average correlation of each gene to the 4 FINs). The red dots indicate the 75 closest neighbors of the GCH1 node in the UMAP shown in Figure (B). Bottom: Gene set enrichment analysis for the top 75 GCH1 closest neighbors, was performed by ranking all the genes based on the correlation shown in the plots above. Normalized enrichment scores (NES) and their *p*-values are indicated. (F) Genes selected for experimental validation from the GCH1 neighborhood, showing their Euclidean distance to the GCH1 node, and their values for the correlation (3 out of the 6) and transcriptomic similarity (all the 7) predictors. The boxplots to the right of each row of the table show where these genes (colored dots) are positioned against the distribution of all 3679 genes in the UMAP.

Thus far, we acquired 13 predictors for each gene, holding values that measure their potential of being ferroptosis suppressors: 6 correlation scores per gene from the correlation module, and 7 transcriptomic similarity scores from the transcriptomic similarity module. To visualize the 13 predictors, we projected them into a plane using the UMAP (Uniform Manifold Approximation and Projection) method (Figure 5A). Each point in this UMAP represents a single gene (3679 total genes, each with 13 predictors values), with adjacent points sharing similar predictor values. We, therefore, could reduce the 13 predictors into a single metric describing the distance between points in the UMAP projection. In parallel, the overall predictor values for each gene were aggregated (Figure S3D, see STAR Methods), and are marked in the UMAP by a blue hue, with a stronger color representing genes with higher predictor values. Among these, are the canonical ferroptosis regulators GCH1, a major ferroptosis suppressor involved in BH4 synthesis,^31^ GPX4,^32^ and CBS, a key regulator of the transsulfuration pathway.^33^

We then focused on GCH1, the 5^th^ gene with the highest average predictor values. We assumed that genes in the GCH1 local neighborhood (Figure 5B; Table S5), would share similar predictor values as GCH1, and therefore could potentially be ferroptosis suppressors. Indeed, many genes cited with ferroptosis related terms in PubMed were found adjacent to the GCH1 node (Figure 5C). Gene ontology enrichment analysis revealed that several genes in the GCH1 neighborhood have oxidoreductase activity (Figure S4A), which can be linked to ferroptosis. To further validate the potential of our prediction, we examined the correlation between all genes in the CCLE dataset to the FIN AUCs (taken from the CTRP drug sensitivity dataset) in pan-cancer (Figure 5D) and to GPX4 dependency score (taken from Achilles dataset, Broad) (Figure 5E). A similar approach was previously used to predict FSP1/AIFM2 as a potential ferroptosis regulator.^34^ Ranking those correlations, we found a positive, significant enrichment of the 75 genes closest to the GCH1 node in the UMAP (Figures 5D and 5E). These results validate the predictive power of the pipeline outcome. Furthermore, we performed the same analysis for all the 1023 possible combinations of predictors (Figure S4B), and found that most of the combinations gave positive, significant enrichment. These results suggest that the GCH1 cluster could be predicted as a potential ferroptosis suppressors regardless of the predictor selection process. Nevertheless, the selected predictors combinations had one of the highest enrichment scores, suggesting that the selection process indeed optimized the results.

### Experimental validation of predicted candidate genes as ferroptosis suppressors

Based on our computation analysis, we predicted that genes in the GCH1 neighborhood would share similar predictor values, and therefore, might suppress ferroptosis. To validate this prediction and highlight the power of the pipeline, we selected several genes from the GCH1 neighborhood for experimental validation. Several considerations were applied for target selection. First and most importantly, we ranked the genes by their Euclidean distance from the GCH1 node in the UMAP (Figure 5C). We filtered out genes which were previously cited in PubMed together with ferroptosis related terms (with few exceptions). To avoid general toxicity, we selected only genes that are not commonly essential in multiple cancer lineages, and therefore their targeting might have fewer side effects. Finally, we prioritized genes which are druggable or ligandable to enable subsequent identification of small molecule inhibitors against those targets.

Among the closest neighbors to the GCH1 node, we proceeded with 7 genes for validation: DECR1, DCXR, NDUFV1, SORD, CANT1, TNFRSF18, and FRAT1. These genes indeed have high values in most of the predictors, especially the correlations of gene expression to AUC (Figure 5F). While the GCH1 neighborhood appeared to be promising, considering the high predictor values of GCH1 (Figure S3D), the GPX4 neighborhood was also examined, given the prominent ferroptotic suppressing activity of GPX4 and its relatively high predictor values (ranked #138 among all genes). Applying similar considerations (such as druggability and toxicity), we selected two additional genes, CTBP1 and PDAP1 from the closest neighborhood of the GPX4 in the UMAP projection (Figures S4C and S4D). As mentioned, most of the 9 selected target genes were not previously associated with ferroptosis (as determined by PubMed search), and are all non-pan essential (Figures S5A and S5B), and druggable targets.

The major consideration in the target selection method is based mainly on the UMAP projection. As the UMAP projection involves a degree of randomness dictated by its initialization seed, we ensured that the genes selection is stable and reproducible regardless the randomness. To this end, we repeated the analysis for 500 UMAP projections, each built with a different random seed (Figure S4E), demonstrating that the selected genes indeed remain in the neighborhood of GCH1 or GPX4. In addition, we further validated that the proximity of the points within the UMAP projection indeed recapitulate proximity in the predictor values (Figure S4F).

**Figure 6:**
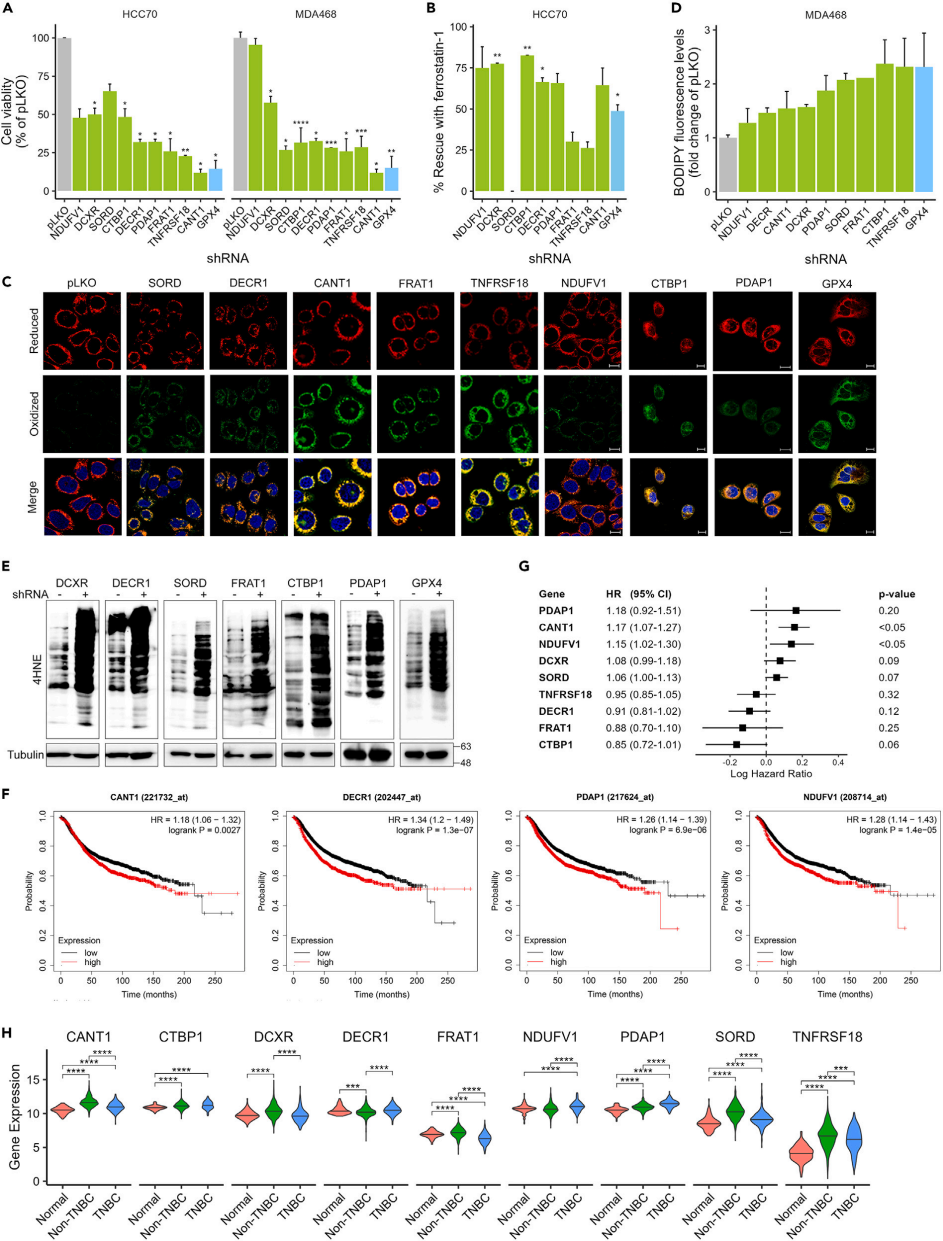
Experimental validation highlighting the ferroptosis inducing potential of gene targeting (A and B) Influence of candidate genes on cell viability. The indicated genes were knocked down (KD) in the indicated TNBC cell lines (HCC70, MDA-MB-468). KD efficiency was assessed by qPCR (Figure S5C), while cell viability was evaluated by MTT assay. Cells were seeded in 96 well plates (50% confluency), without (A) or with Ferrostatin (5 μM) (B), and 72 h later, cell viability in the control (pLKO) or genes KD cells was measured. Percent of cell viability (A) and percent rescue with Ferrostatin (B) are shown (mean ± SD of at least two independent experiments). KD of GPX4 was used as a positive control. P-values were measured by one-sample t-test (∗*p*-value<0.05, ∗∗*p*-value<0.01, ∗∗∗*p*-value<0.001, ∗∗∗∗*p*-value<0.0001). (C and D) Knock-down of the indicated genes was performed in MDA-MB-468 cells by shRNA. Bodipy-C11 staining was used to visualize lipid peroxidation. Representative confocal images are shown (C) (scale bar, 10μm). Fluorescence measurements are given in (D). pLKO (empty vector) and shGPX4 were used as a negative and positive control, respectively. In (D), means ± SD are shown of two repeats. (E) Western blot analysis of 4HNE-adduct, for MDA-MB-468 in which the indicated genes were knocked down. Tubulin was used as a housekeeping gene. (F) Kaplan-Meier plots for genes with significant differences in prognosis. (G) Hazard ratios (HR) were calculated for the indicated genes in patients with breast cancer (MetaBric data, *n* = 1904 patients) using the Cox proportional hazards model. (H) Expression of the indicated genes in patients, taken from TCGA data (*n* = 114 normal, 879 non-TNBC, and 180 TNBC).

For the experimental validation, the 9 selected genes were knocked down by shRNAs in MDA-MB-468 and HCC70 basal-like TNBC cell lines (Figure S5C), and their influence on ferroptotic death was examined by characteristic assays.^35^ As shown in Figure 6A, knocking down these genes significantly reduced cell viability in the two TNBC cell lines, with the exception of NDUFV1 in MDA-MB-468 cells. In most cases, the inhibitory effect on cell viability was rescued by the ferroptosis inhibitor, ferrostatin (Figure 6B). Next, we assessed the effect of the genes KD on lipid peroxidation using the fluorescent sensor BODIPY-C11. The increased levels of the oxidized form of the sensor upon genes knockdown are demonstrated by representative confocal images (Figure 6C), along with the quantification of the fluorescence intensity (Figure 6D). This increase in lipid peroxidation was accompanied with enhanced 4HNE (4-Hydroxynonenal) levels shown in the Western blots (WB) in Figure 6E. In those experiments, GPX4 targeting by shRNA was used as a positive control. Importantly, one of the selected genes in the GPX4 neighborhood, PDAP1 (PDGFA-associated protein 1), was identified independently in our recent study using a different approach.^23^ We showed that the depletion of PDAP1 not only induced ferroptosis in basal-like breast cancer cells *in vitro* but also induced ferroptosis in basal-like breast tumors in a xenograft mouse model and consequently inhibited tumor growth,^23^ suggesting that the identified ferroptosis suppressors could be used for cancer therapy. Four of the genes (CANT1, DECR1, PDAP1, NDUFV1) had significant prognostic effects, as determined by Kaplan-Meier analysis using relapse-free survival data (Figure 6F). Two of these genes, CANT1 and NDUFV1, were also prognostic using overall survival data as determined by Cox analysis (Figure 6G). The expression level of most of the genes is high in TNBC tumors (Figure 6H), further suggesting that their targeting could be beneficial for TNBC therapy.

Overall, we validated 9 potential ferroptosis repressors in TNBC, many of which were shown to have an inhibitory effect in BC upon targeting (Table 1). While these genes may enhance ferroptosis through different mechanisms, we found some common gene ontologies, including oxidoreductase activity, which might be associated with many ferroptosis regulators (Figure S5D).

**Table 1:** Key effects of validated genes in breast cancer (BC) or other cancer types

Gene	Effect in cancer	Reference
CANT1	Prognostic factor, promotes cell proliferation and invasion in lung adenocarcinoma	Yao et al.^36^
CTBP1	Elevated in BC, targeting increases sensitivity to chemotherapy	Birts et al.^37^, Deng et al.^38^
DCXR	Promote BC cell proliferation. Targeting inhibits tumor growth *in vivo*	Jin et al.^39^
DECR1	Targeting induces ferroptosis in prostate cancer *in vitro* and *in vivo*	Nassar et al.^40^
FRAT1	Highly expressed in TNBC	Nam et al.^41^
NDUFV1	Increases BC proliferation, however targeting increases the metastatic ability	Kim and Singh^42^, Santidrian et al.^43^
PDAP1	Targeting induces ferroptosis in BC; targeting inhibits tumor growth *in vivo*	Vinik et al.^23^

## Discussion

In this study, we established a powerful computational framework applying a unique approach of integrating the correlation and the transcriptomic similarity modules to identify suppressors of cell-death pathways as candidate targets for cancer therapy. Considering the vulnerability of TNBC to ferroptosis and the lack of effective therapeutic targets for patients with TNBC, we focused on ferroptosis as the targeting death pathway.

The integrated pipeline relies on (i) the correlations between gene expression/essentiality and ferroptosis sensitivity, which yielded 6 predictors per gene, and (ii) the similarity between the transcriptomic response of gene targeting and the transcriptomic response to FINs, which yielded 10 predictors (Figure 1). To reduce the variability associated with the diverse transcriptomic response to different FINs, we reduced the number of the transcriptomic similarity predictors, by evaluating the similarities between the 10 profiles and filtering out the transcriptomic profiles most dissimilar to the others. This analysis enabled the reduction of the transcriptomic similarity predictors from 10 to 7, which were eventually combined with the 6 correlation predictors using dimensionality reduction by UMAP. We assumed that the proximity in the UMAP of a gene to canonical ferroptosis repressors (mainly GCH1 and GPX4) is the best metric in the selection process of ferroptosis suppressors. Indeed, experimental validation of 9 genes (7 genes in GCH1 proximity, and 2 from GPX4 neighborhood) demonstrated the predicting power of the pipeline, as their knockdown enhanced lipid peroxidation and reduced TNBC cell viability (Figure 6). Hence, we established a powerful resource for the identification of candidate ferroptosis suppressors.

A major challenge in pipeline optimization was associated with predictor selection, which was a necessary process due to the high variability in the transcriptomic responses to various FINs in different biological models and time points.^44^ To overcome the high transcriptomic variability, we selected the transcriptomic similarity predictors unbiasedly by assessing their transcriptomic similarity to each other (Figure 4D). We validated the selection process by text-mining analysis of all the possible combinations of predictors (1023 in total) (Figure 4E). This analysis highlights the statistical significance of the predictor selection process and suggests that the text-mining can be used by itself as a method for predictor selection.

Importantly, we also demonstrated that the GCH1 neighborhood (Figure 5B) is statistically significantly enriched with potential targets for ferroptosis (Figures 5D and 5E). A similar analysis for all the possible predictor combinations (Figure S4B) suggests that while the predictor selection step can indeed optimize the results, the pipeline has the potential to predict ferroptosis suppressors regardless of this step. This is an important observation, which highlights the potential of generalizing the pipeline to other death pathways. Furthermore, we showed that the distances between points on the UMAP projection significantly correlate with the distances in the predictor matrix (Figure S4F), confirming the hypothesis that genes with similar predictor values are clustered together. Finally, we made sure that the output of our pipeline is stable for randomness (Figure S4E) considering that the UMAP is a stochastic algorithm. Taken together, we established a statistically significant algorithm that could accurately predict ferroptosis repressors as potential candidates for cancer therapy.

The identified candidate genes may impose their ferroptosis suppression through major known ferroptosis axes,^45^ including the GSH-GPX4 axis, the FSP1-CoQ10 axis,^7^^,^^46^ the GCH1-BH4 axis,^6^ the DHODH-CoQH2 axis,^8^ or by as of yet unknown mechanism. Among the 9 genes that were experimentally validated, DECR1 was previously shown to protect prostate tumor cells from ferroptosis, mainly through the regulation of PUFA oxidation.^40^ DECR1 was positioned very closely to the GCH1 node, and exhibited high predictor values, similar to GCH1. DCXR was also placed in close proximity to GCH1, had high predictor values, and was found to be the most correlating gene to erastin AUC (Figure 2D). However, its involvement in ferroptosis has not been reported so far. DCXR plays a role in xylitol metabolism,^47^ which in turn can affect glutathione levels,^48^ but its role in ferroptosis might not be directly related to the GCH1-BH4 axis and requires further investigation. CTBP1, one of the GPX4's closest neighbors, was also validated to be a good target for ferroptosis induction. This gene, a metabolic sensor of redox status, was recently found to be involved in ferroptosis.^49^ Another neighbor of GPX4 is PDAP1, which we recently identified as a ferroptosis repressor and demonstrated its potential as a target for TNBC therapy in xenograft TNBC mouse model.^23^ Importantly, we showed that PDAP1 depletion induced ferroptotic death both *in vitro* and *in vivo*, possibly through the AKT-mTOR-SREBP1-SCD1 axis, further demonstrating the power of the described pipeline and the potential of the discovered candidate targets for cancer therapy.

It is important to note that SLC7A11, the actual target of erastin, was ranked very low in this pipeline, suggesting that the output of our approach can produce valid ferroptosis inducing targets, but not all possible targets will be ranked high in the pipeline. In the case of SLC7A11, this can be explained by a negative correlation of its expression to FINs AUCs, which is counter intuitive. While SLC7A11 correlated with FIN AUCs pan-cancer (as shown in Figure 5D), its correlation specifically in breast cancer is poor (also when comparing to CTRP data).

Notably, the CMAP approach was previously used to identify drugs that increased the expression of ferroptosis related genes,^50^^,^^51^ and uncovered a connection between ferroptosis and HDAC inhibition.^52^ Correlations of gene essentiality to ferroptosis susceptibility identified FSP1 as a major ferroptosis regulator.^34^ The integration of both methods was proven to be highly beneficial. The integration mode increased the confidence in target selection and reduced the rate of false positives, which are common in many correlation-based drug discovery methods. The described pipeline, combining the correlations to FINs sensitivity together with transcriptomic similarities, followed by the dimension reduction and ranking potential targets by their proximity to major nodes, can potentially be streamlined to produce a computational pipeline revealing new targets to any desired phenotype induced by specific drugs, and not only to ferroptosis. Such a pipeline will require vulnerability data of cancer cells to the drug, and their transcriptomic response, and will produce a ranked list of genes, from which the targets can be selected based on other pharmacological considerations, such as novelty, general toxicity, and druggability. Hence, it can provide a powerful computational tool that can be used to discover targets of many different pathways affecting cancer growth, progression, and patient survival.

### Limitations of the study

While the established pipeline has the potential to successfully predict suppressors of different cell-death pathways beyond ferroptosis, we validated its potency only for ferroptosis in the scope of this study. Further target validations of other death modules, in future projects, would greatly increase the applications of this pipeline. For other pathways, we propose to improve the predictor selection process for the pathways exhibiting highly variable predictors. In such pathways, a predictor selection process should be performed based on the predictors’ variance as described in this study and validated with text-mining or with another suitable method. Importantly, for each selected death pathway, the pipeline requires pre-existing information on the pathway in question to assign pathway nodes, such as GCH1 and GPX4 for ferroptosis. Eventually, it would be beneficial to predict death-pathway suppressor candidates based solely on the predictor values, rather than the proximity to major nodes.

## Resource availability

### Lead contact

Further information and requests for resources and reagents should be directed to and will be fulfilled by the Lead Contact, Sima Lev (Sima.Lev@weizmann.ac.il).

### Material availability

This study did not generate new unique reagents.

### Data and code availability

• RNAseq data have been deposited at GEO and are publicly available as of the date of publication. Accession numbers are listed in the key resources table. This article also analyzes existing, publicly available data. These accession numbers for the datasets are listed in the key resources table.
• The code written for this article is available in Github (https://github.com/SimaLevLab/Pipeline-for-discovery-of-ferroptosis-targets), and was also deposited at Zenodo. The code is publicly available as of the date of publication. DOI is listed in the key resources table.
• Any additional information required to reanalyze the data reported in this article is available from the lead contact upon request.

## Acknowledgments

Sima Lev is the incumbent of the Joyce and Ben B. Eisenberg Chair of Molecular Biology and Cancer Research. We thank Itay Tirosh for reviewing the article, and Sven Lindermann, Klaus Urbahns and other 10.13039/100004334Merck members for their valuable input throughout the project.

This study is supported by 10.13039/100009945Merck KGaA, Darmstadt, Germany.

## Author contributions

YV designed and performed the computational analysis and wrote the article. AM designed and performed the experiments in Figure 6. HR and VD prepared the RNA for RNAseq and performed the experiment shown in Figure S1A. FG and DW contributed to the discussion and reviewed the article. SL supervised the study, contributed to the analysis and interpretation of the data, ensured financial support, and wrote the article.

## Declaration of interests

Authors declare that they have no competing interests.

## STAR★Methods

### Key resources table

**Table:** 

REAGENT or RESOURCE	SOURCE	IDENTIFIER
**Antibodies**		

anti-4-HNE	Sigma	AB5605; RRID: AB_569332
Anti-beta-Tubulin	Cell signaling	2146; RRID: AB_2210545

**Chemicals, peptides, and recombinant proteins**		

Erastin	Sigma	E7781
RSL3	Sigma	SML2234
FIN56	Sigma	SML1740
Ferrostatin-1	Sigma	SML0583

**Critical commercial assays**		

C11-BODIPY 581/591 fluorescence reporter	Cayman	27086

**Deposited data**		

RNA-seq for MDA-MD-468 and HCC70	this paper	GEO: GSE235201
RNAseq for SUM159, HS578 and MDA-MB-231	this paper	GEO: GSE255459
CMAP signature dataset	Broad Institute	GEO: GSE92742
CCLE dataset, version 20Q4	DepMap project	https://depmap.org/portal/
Achilles dataset, version 21Q1	DepMap project	https://depmap.org/portal/
FerrDB version 1		http://www.zhounan.org/ferrdb/legacy/index.html

**Experimental models: Cell lines**		

MDA-MB-231	ATCC	RRID: CVCL_0062
MDA-MB-436	ATCC	RRID: CVCL_0623
SUM159	ATCC	RRID: CVCL_5423
HS578T	ATCC	RRID: CVCL_0332
BT549	ATCC	RRID: CVCL_1092
MDA-MB-468	ATCC	RRID: CVCL_0419
BT20	ATCC	RRID: CVCL_0178
HCC1937	ATCC	RRID: CVCL_0290
HCC70	ATCC	RRID: CVCL_1270
SKBR3	ATCC	RRID: CVCL_A2GI
JIMT1	ATCC	RRID: CVCL_2077
T47D	ATCC	RRID: CVCL_0553
HCC1143	ATCC	RRID: CVCL_1245
MDA-MB-453	ATCC	RRID: CVCL_0418
MCF-7	ATCC	RRID: CVCL_0031
BT474	ATCC	RRID: CVCL_0179
HEK293T	ATCC	RRID: CVCL_0063

**Oligonucleotides**		

Primers for qRT-PCR experiment, see Table S6	this paper	Table S6

**Recombinant DNA**		

pLKO-SORD	Sigma	TRCN0000028052
pLKO-FRAT1	Sigma	TRCN0000062463
pLKO-NDUFV1	Sigma	TRCN0000025872
pLKO-CANT1	Sigma	TRCN0000051898
pLKO-TNFRSF18	Sigma	TRCN0000058373
pLKO-DECR1	Sigma	TRCN0000046514
pLKO-DCXR	Sigma	TRCN0000038955
pLKO-CTBP1	Sigma	TRCN0000285086
pLKO-PDAP1	Sigma	TRCN0000299988
pLKO-GPX4	Sigma	TRCN0000046252

**Software and algorithms**		

R version 4.4.0	https://www.r-project.org/	
RStudio version 2024.04.2	Posit Software	
KMplotter	https://kmplot.com/analysis/	
ZEN imaging software, blue edition, version 3.2	Zeiss	
Code generated in this study	This paper	https://doi.org/10.5281/zenodo.13370885

### Experimental model and study participant details

#### Cell lines

The breast cancer cell lines MDA-MB-231, MDA-MB-436, SUM159, HS578, BT549, MDA-MB-468, BT20, HCC1937, HCC70, SKBR3, JIMT1, T47D, HCC1143, MDA-MB-453, MCF-7, BT474 (all of female origin) and the human embryonic kidney (HEK) 293T cells were originally obtained from the American Type Culture Collection (USA). The cells were grown in RPMI (all breast cancer lines) or DMEM (HEK293 cells) containing 10% fetal bovine serum (Gibco BRL, USA) and penicillin/streptomycin. Cells were cultured at 37°C in a humidified incubator of 5% CO_2_. Cell lines were routinely (once a month) checked for mycoplasma using a commercially available kit (Biological Industries, Israel).

### Method details

#### Measurement of cell sensitivity to ferroptosis

Breast cancer cell lines were seeded in 96-wells plate (50% confluency), and 24 hr later were treated with erastin, RSL3 or FIN56 using different doses. Cell viability was measured 72 hr later by MTT assay (M2128, SIGMA). Cell viability (taken as % of control untreated (DMSO) cells) was plotted against drug concentration, and dose response curves were fitted to the data by the drm function from the drc package in R. AUCs (area under the curves) were extracted using the computeAUC function from the PharmacoGx package in R.

#### Generation of the correlation scores

Pearson’s correlations between the AUCs of the 3 FINs (acquired from the dose response curves) and gene expression levels (extracted from the CCLE data, 20Q4 version) and gene dependency scores (extracted from the Achilles dataset, 21Q1 version) was performed using the cor function in R. CCLE and Achilles datasets were downloaded from DepMap. Venn diagrams showing the genes with high and low correlations in all 3 FINs were done using the VennDiagram package.

#### RNA sequencing

Five TNBC cell lines (MDA-MB-468, HCC70, BT549, SUM159, Hs578) were treated with erastin (9 hours) and RSL3 (4 hours) using the IC_50_ concentrations which were deduced from the dose response curves created above. DMSO was used as control. Total RNA was extracted using TRI Reagent (Sigma-Aldrich), and its quality was assessed using Agilent 4200 TapeStation System (Agilent Technologies, Santa Clara, CA). RNA-seq libraries were generated by applying a bulk adaptation of the MARS-seq protocol, as previously described.^53^ Libraries were sequenced by the Illumina Novaseq 6000 using SP mode 100 cycles kit (Illumina). Mapping of sequences to the genome and generation of the count matrix was performed by the UTAP pipeline (Weizmann Institute). Libraries normalization, filtration of low count genes and discovery of differentially expressed genes was performed using the edgeR and Limma packages in R. PCA plot was generated using the factoextra package. UpSet plot was generated using the ComplexUpset package.

#### Validation of the ferroptosis response signature

19 public datasets, representing the transcriptomic response of ferroptosis induction in different cell lines and models, were downloaded from GEO (Table S3). In each dataset, the log2 fold changes in gene expression between the inducer and its control were calculated using Limma. The enrichment, in those datasets, of genes upregulated by FINs, was measured using the ssGSEA method, implemented by the gsva function from the GSVA package in R. The top 20 ranking genes were uploaded to the connectivity map site (https://clue.io/). The results were analyzed using the campR package in R.

#### Generation of transcriptomic similarity scores

The CMAP signature dataset was downloaded from GEO (accession number GEO: GSE92742). The level 5 signatures were downloaded, together with all relevant meta-data. The signatures were filtered to include only the consensus shRNA signatures (trt_sh.cgs). From each signature, the top 20 and bottom 20 genes were taken to form the upregulated and downregulated gene sets associated with that signature. The enrichment of all those gene sets in our 10 treatment points was performed by the Camera method from the Limma package in R.^30^ In CMAP, each shRNA was tested in several cell lines – the scores for each shRNA were aggregated into one score by taking the maximum value of the original scores. The resulting scores were then ranked. For each shRNA, the final transcriptomic similarity score was calculated to be the ranked score of the upregulated set minus the ranked score of the downregulated set. shRNAs with higher score are therefore considered to induce transcriptomic response similar to those of the FINs, i.e. their target genes have a potential to be ferroptosis suppressors.

As a filtration step for the transcriptomic similarity scores, we adapted a method from Smith et al.,^54^ by which we measured the similarities between the 10 transcriptomic profiles obtained from the RNAseq. In brief, we calculated the Pearson correlation for every pair of transcriptomic profiles, using the log2 fold changes for all genes in each profile. For each profile, the 9 correlation scores to the other 9 profiles were summed up, excluding any negative correlations or the correlation of each profile to itself. These summations were normalized on a scale from 0 to 100. This normalized metric represents the similarity of each transcriptomic profile to their average profile.

#### PubMed search for ferroptosis publications

For given sets of genes (the top correlating genes to the FIN AUCs, or the GCH1 closest neighbors), we performed a PubMed search with terms related to ferroptosis (“Ferroptosis”, “Gluthathione”, “GPX4”, “Iron”, “Lipid Peroxidation”, “SLC7A11”). This search was automated using the rentrez package in R, and is updated for November 2023. In our method for validation of the predictor selection method, we generated gene sets as follows: (i) Predictor combination sets: we combined the 6 correlation module predictors, together with any combination of the 10 transcriptomic similarity predictors (in total, $\sum_{k=1}^{10}\frac{10!}{k!\left(10-k\right)!}=1023$ combinations). For each such combination, we took the top 50 genes with the highest predictor values. (ii) Random sets: 2000 sets of 100 randomly selected genes each. Each of those 3023 sets were analyzed for citations with the same terms in PubMed. Due to the large number of sets, for this analysis we used the Geneshot tool.^55^

#### Combining the predictor scores

The transcriptomic similarity and correlation predictors were normalized and used to define a single metric that will predict genes with ferroptosis suppressor activity. The UMAP method was used to project the transcriptomic similarity and correlation scores of all genes on a 2D plane. The UMAP was implemented using the UMAP function in R, setting the “min_dist” parameter to 0.05 and keeping the rest of the parameters as default. The prediction metric was determined to be the Euclidean distance in the UMAP projection of each gene from a central node, selected for being a major ferroptosis suppressor (such as GCH1). In another approach, we averaged all the predictor values as follows: We calculated the average of the 3 correlation scores between gene expression levels and the 3 FINs AUCs, as well as the average of the 3 correlation scores between gene dependency scores and the 3 FINs AUCs. Then, the maximum average of the two was taken. This maximum value was ranked, and averaged with the ranking of the average of all transcriptomic similarity scores. This measure was highlighted on the UMAP in blue scale. In addition, we measured the distance of the genes from each other by creating a distances matrix based on the normalized predictor values, and compared those distances to the distances measured on the UMAP, to validate that local neighborhoods in the UMAP indeed correlates with actual similarities in predictor values.

To validate the hypothesis that the genes closest to the GCH1 node in the UMAP have ferroptosis suppression potential, the pan-cancer CCLE data (version 20Q4), Achilles data (version 21Q1) and CTRP drug response data were downloaded from Depmap portal (https://depmap.org/portal/). Gene expression levels were correlated to the GPX4 dependency score (taken from the Achilles dataset) and to FINs AUCs (Erastin, RSL3, ML210, ML162, taken from the CTRP dataset). To verify that the selected set of genes (GCH1 node neighbors) are among the highest correlating genes in both cases, we performed gene set enrichment analysis, using the fgsea function from the fgsea package in R. In the fgsea function, the enrichment score of the selected genes was measured against a ranked list of the genes, ranked by the above correlations of expression to GPX4 essentiality, and expression to FINs AUCs. The same analysis was also done on all 1023 possible combinations of predictors; for each such combination a UMAP was built and the top 75 genes with the closest Euclidean proximity to GCH1 were analyzed in the same way.

#### Gene knockdown

Lentiviral vectors encoding shRNAs of the validated genes were purchased from Sigma. The catalog number of the shRNAs used are: SORD (TRCN0000028052), FRAT1 (TRCN0000062463), NDUFV1 (TRCN0000025872), CANT1 (TRCN0000051898), TNFRSF18 (TRCN0000058373), DECR1 (TRCN0000046514), DCXR (TRCN0000038955), CTBP1 (TRCN0000285086), PDAP1 (TRCN0000299988) and GPX4 (TRCN0000046252). 3rd generation lenti-viruses were produced in HEK293T cells by co-transfecting the pLKO.1-shRNA constructs with pLP (pVSVG), pLP1 (pMDL) and pLP2 (pREV) expressing the virus envelope protein. Twelve hours later, the medium was changed and viruses were collected 24 and 48 h later. Viral supernatants were filtered through 0.45 μm pore size filters and stored at -80°C. Cells were infected with the viruses in the presence of 8 μg/ml polybrene for 24 h and then subjected to selection with media containing 1 μg/ml puromycin for 72-96 h. Cells infected with the empty virus were used as control. Following knockdown, cell viability assay was performed using MTT as described above, in the presence of the ferroptosis inhibitor Ferrostatin-1 (5μM) or DMSO as control.

#### Lipid peroxidation measurement

Lipid peroxidation in live cells was detected by the C11-BODIPY 581/591 fluorescence reporter (#27086, CAYMAN). Cells were plated in High-Content Imaging Glass Bottom 96-well Microplates (Cellvis) for ∼24 hr and then treated with the indicated drugs. The lipid peroxidation sensor C11-BODIPY (581/591) (7 μM) was added for 30-40 min together with 1 mM Hoechst 33342 (Sigma-Aldrich) in regular RPMI full media. Cells were gently washed twice with PBS and incubated in live cell imaging solution (Invitrogen, A14291DJ). Fluorescence was measured at 581/590 nm (excitation/emission) for the reduced dye, and at 488/510 nm (excitation/emission) for the oxidized dye, while Hoechst was measured at 350/461 nm (excitation/emission) using the Infinite 200 PRO Tecan microplate reader (Tecan Inc., Switzerland). The green-to-red fluorescence intensity ratio was used to measure lipid peroxidation. The values were normalized to cells number in each well using Hoechst staining values. Confocal microscopy images of live cells stained with C11-BODIPY were acquired using the LSM800 (Zeiss), 40X oil lens and the ZEN Imaging Software (Zeiss).

Lipid peroxidation was also estimated by the level of 4-HNE (4-hydroxynonenal) protein adducts as detected by anti-4-HNE antibody (AB5605, SIGMA) in western blot analysis. For this experiment, cells were lysed in lysis buffer containing 0.5% Triton X-100, 50 mM Hepes pH 7.5, 100 mM NaCl, 1 mM MgCl_2_, 50 mM NaF, 0.5 mM NaVO_3_, 20 mM β-glycerophosphate, 1 mM phenylmethylsulfonyl fluoride, 10 μg ml^−1^ leupeptin, and 10 μg ml^−1^ aprotinin. Cell lysates were centrifuged at 14,000 rpm for 15 min at 4°C. Protein concentration of the supernatants was measured by Bradford assay (Bio-Rad, Hercules, CA). Equal amounts of total protein (30–50 μg per sample) were analyzed by SDS–PAGE (polyacrylamide gel electrophoresis) and Western Blotting was performed. Anti-Tubulin antibody (Cell Signaling, 2146) was used as loading control.

#### RNA extraction and real time PCR

Total RNA from cell lines was extracted and purified using TRI Reagent (Sigma-Aldrich). RNA was reverse-transcribed into complementary DNA (cDNA) using the High-Capacity cDNA Reverse Transcription Kit (Applied Biosystems; Cat. No. 4368814) with random primers according to the manufacturer’s instructions. Real-time PCR analysis was performed in QuantStudio-3 Real-Time PCR system (Applied Biosystems, Thermo Fisher Scientific) using SYBR Green Master Mix reagents (Roche) according to the manufacturer’s guidelines. House-keeping gene β-actin was used for normalization. The relative levels of mRNA were calculated using the ΔΔCT method. Primer sequences are listed in Table S6.

#### Patient dataset analysis

Expression levels of genes of breast cancer patients was taken either from the METABRIC or TCGA datasets. Univariate Cox regression was done using the coxph() function from the `survival` package, using the overall survival follow-up data included in the MetaBric dataset. Gene ontology analysis was performed by the gost() function in the gProfiler2 package in R. Kaplan-Meier plots were performed using KMplotter,^56^ using relapse-free survival as the event, and with automatic selection of expression cutoff (in total, 2032 patients examined).

### Quantification and statistical analysis

The statistical test used to determine the significance levels for each experiment was done in R and is described in the figure legends. In most cases (unless otherwise mentioned in the figure legends), t-test was used to measure statistical significance. *P*-values lower than 0.05 were considered as statistically significant.

## Supplemental information

Document S1. Figures S1–S5 and Tables S3 and S6

Table S1. Correlation of AUCs to gene expression and essentiality, related to Figure 2

Table S2. Differentially expressed genes based on FIN induced TNBC lines, related to Figure 2

Table S4. Connectivity map enrichment scores, related to Figure 4

Table S5. Gene list - GCH1 nearest neighbors, related to Figure 5
