# Supplementary material for: Computational pipeline predicting cell death suppressors as targets for cancer therapy
Source: iScience. 2024 Aug 30;27(9):110859. doi: 10.1016/j.isci.2024.110859 (PMC11416655; doi:10.1016/j.isci.2024.110859)
Supplement: Document S1. Figures S1–S5 and Tables S3 and S6 [file mmc1.pdf]

iScience, Volume *27*

Supplemental information

Computational pipeline predicting cell death suppressors as targets for cancer therapy

Yaron Vinik, Avi Maimon, Harsha Raj, Vinay Dubey, Felix Geist, Dirk Wienke, and Sima Lev

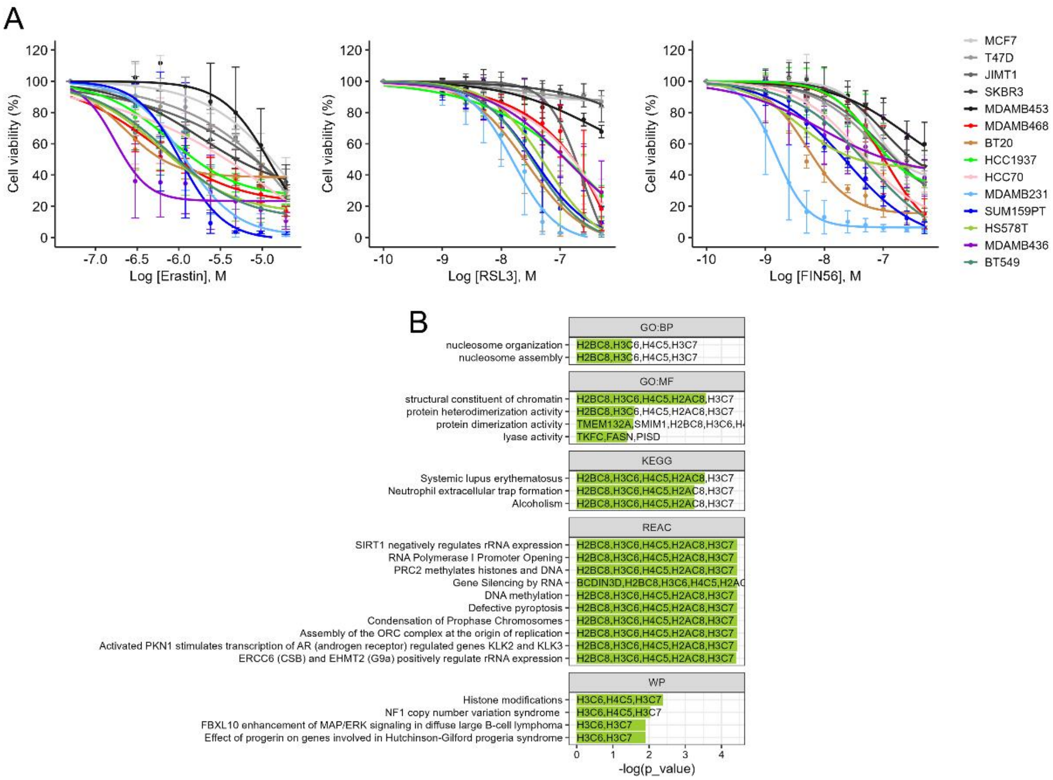

Figure S1. Supporting information for extraction of correlation scores (related to Figure 2) **(A)** The indicated breast cancer lines were seeded in 96 well plates (maintaining a confluency of ~50%). 24 hours later, the indicated FIN (erastin, RSL3, FIN56) were added in different concentrations. 72 hours later MTT was performed to assess the level of cell viability. Plots show means ± sd of 3-4 independent repeats. **(B)** Gene ontology and pathway enrichment for the 41 genes with the highest correlation between gene expression and FIN AUCs.

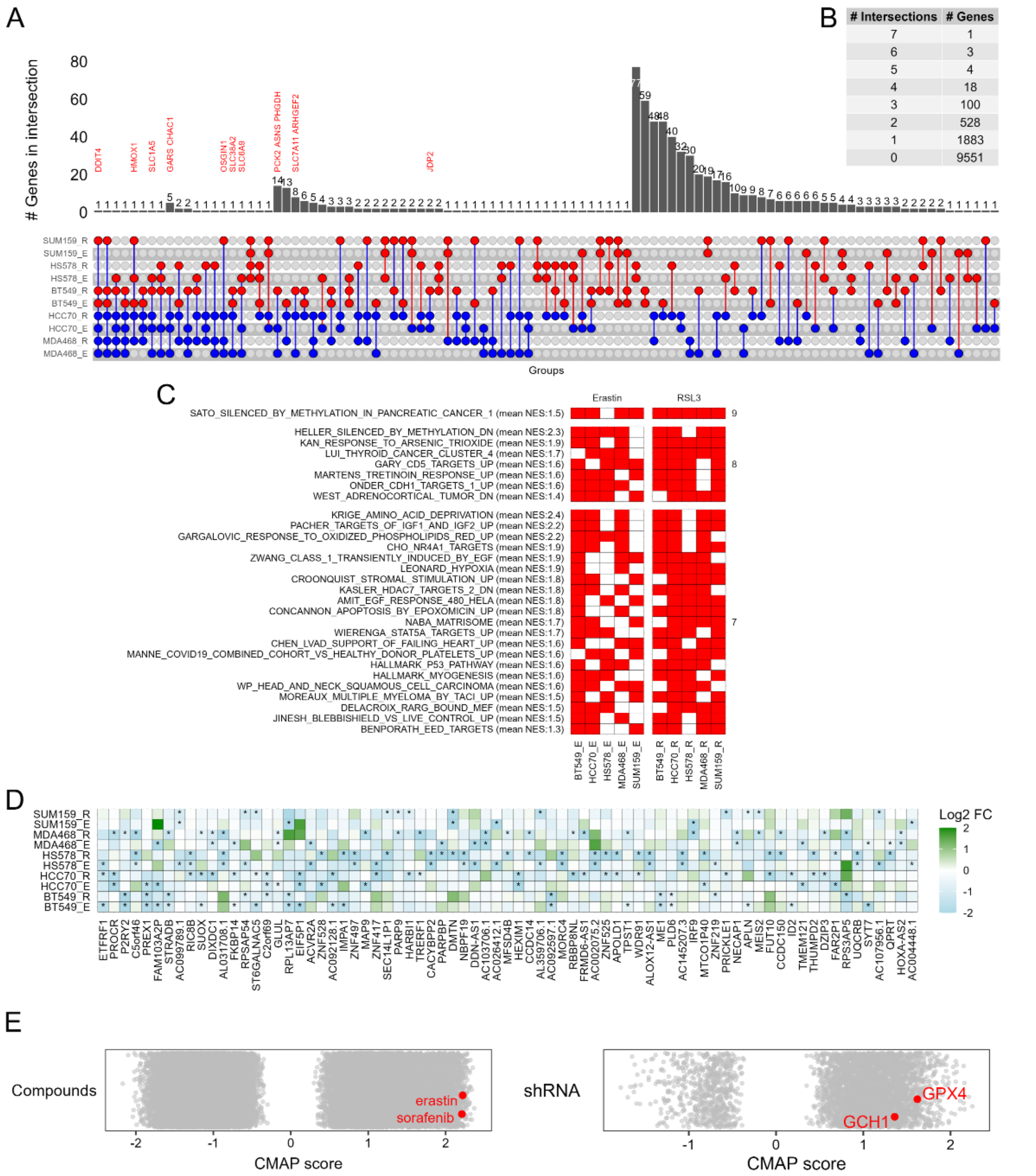

Figure S2. Supporting information for the ferroptosis response signature (related to Figure 3)

(A) UpSet plot showing the number of significant (p-value < 0.05) differentially expressed genes (DEGs, top bars) for any intersection of treatments (bottom plot). In the bottom plot, the blue and red points indicate basal and mesenchymal cell lines, respectively, and the gray and white shaded lines indicate treatment with erastin and RSL3, respectively. The annotated genes are ferroptosis to apoptosis biomarkers found in our previous publication. **(B)** Summary of the number of DEGs per number of treatments in each intersection. **(C)** Gene set enrichment analysis was performed for the 10 treatments. The pathways with the highest average normalized enrichment scores (NES)

are grouped by the number of treatments in which this pathway is significant (indicated by a red square) among the 10 treatments. **(D)** Heatmap showing the 75 genes with the lowest fold change, i.e. the genes most downregulated in response to erastin and RSL3. **(E)** The top 20 genes of the heatmap shown in Fig. 3C were used to delve the connectivity map database of signatures, finding compounds (left) and shRNA (right) which induce transcriptomic profile enriched for those 20 genes.

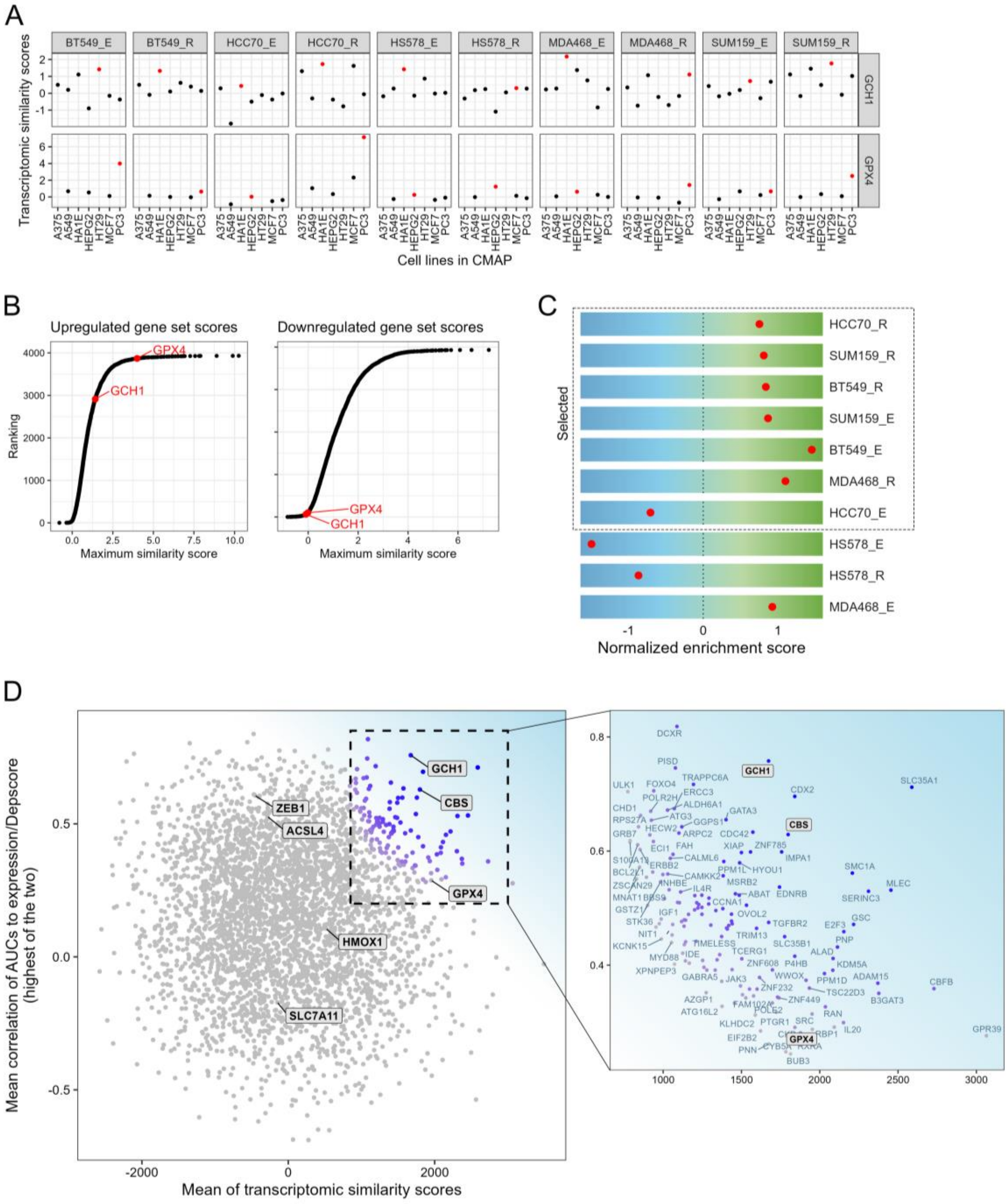

Figure S3. Calculating the ranked transcriptomic similarity scores per gene (related to Figure 4)

(A) Similarity scores for each gene and each treatment were calculated across the cell lines available from the CMAP dataset. This representative figure shows the scores for the upregulated set of 20 genes, extracted from the signatures of GPX4 and GCH1, as an example. In each panel, the red dot indicates the maximum value. The similarity score for each gene per treatment were taken to be this maximum value. **(B)** The maximum values from A were ranked. Representative plot showing the ranks of the scores are taken from the “BT549_E” treatment. **(C)** The final transcriptomic similarity scores for each gene were calculated by subtracting the downregulated gene set score (shown in B, right panel) from the upregulated gene set scores (shown in B, left panel). The red dots indicate the enrichment of 40 known ferroptosis suppressors, measured by

GSEA. **(D)** Scatterplot showing the aggregated predictor values for all genes. The x-axis shows the average of all 7 transcriptomic similarity predictors. The y-axis shows the average of the 3 correlations of gene expression to AUCs, or the average of the 3 correlations of gene essentiality to AUCs (the higher of the two). The upper-right corner, including the genes with the highest predictor values, is zoomed-in on the left.

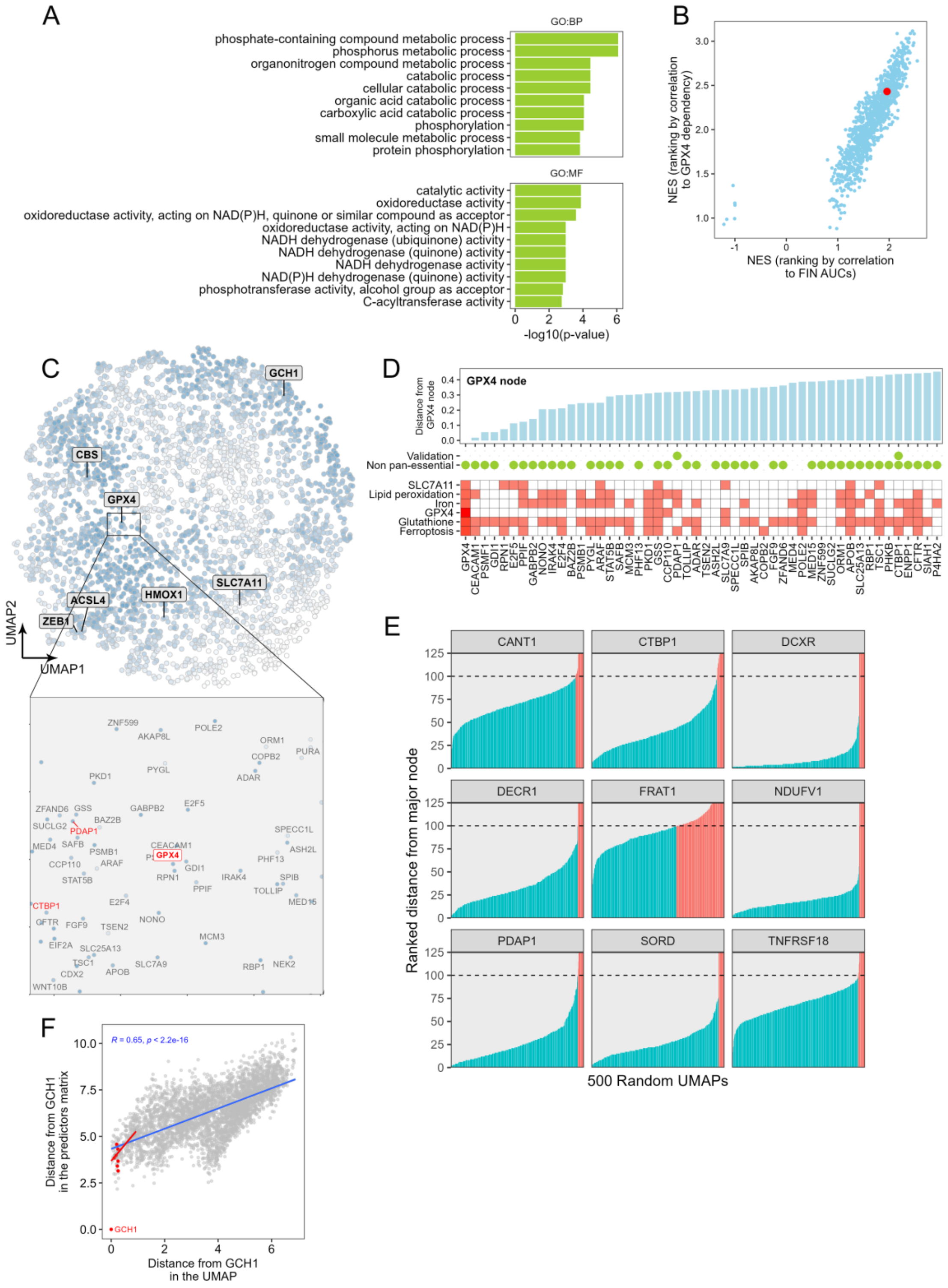

Figure S4. Integrating the correlation and transcriptomic similarity predictors for predicting ferroptosis targets (related to Figure 5)

(A) Gene ontology (biological processes [top], molecular functions [bottom]) was performed for the GCH1 neighborhood genes shown in Fig. 5B. Only significant GO terms are shown. **(B)** 1023 combinations of predictors (see methods) were observed. For each combination, UMAP projection

was performed, and the top 75 genes closest to the GCH1 node were selected for further analysis as described in Fig. 5D-E. In brief, all the genes were ranked by the correlations of their expression levels to the dependency score of GPX4 or to the AUCs of 4 FINs taken from the CTRP dataset. The enrichment of the 75 GCH1-neighboring genes in each combination was measured by GSEA against all the genes ranked by those correlations, and the normalized enrichment scores (NES) are shown in this plot. The red dot indicates the actual set of predictors used for the UMAP shown in Figure 5B (note, the NES values in Fig. 5D-E are a bit different compared to the NES of the red dot in this analysis, due to the number of signatures examined in the GSEA in each case). **(C)** Zoom-in of the GPX4 node in the UMAP from Fig. 5A. **(D)** The genes in the GPX4 neighborhood were ranked by their proximity to GPX4. PubMed search of each gene with 6 ferroptosis related terms was performed. A red labeled box indicates a citation for the gene with the indicated term in PubMed. **(E)** The UMAP projection shown in Figure 5A was recreated 500 times, each time with a different random seed for the UMAP creation (the seeds themselves were also randomly selected). For each UMAP, the distance between the indicated genes (which were selected for experimental validation) and the GCH1 node (for most genes) or the GPX4 node (for CTBP1 and PDAP1) was measured and ranked among other genes. **(F)** Correlation between the distance on the UMAP projection compared to the distance of the predictor matrix. Each point represents a gene. The x-axis shows the Euclidean distance of each gene from GCH1 in the UMAP shown in Fig. 5A. The y-axis shows the Euclidean distance between each gene and GCH1, calculated using the normalized values of the 13 predictors themselves. The blue line represents the linear correlation for the entire data; the red line shows the linear correlation for the 200 genes closest to GCH1. The red points indicate the genes from the GCH1 local neighborhood that were chosen for experimental validation.

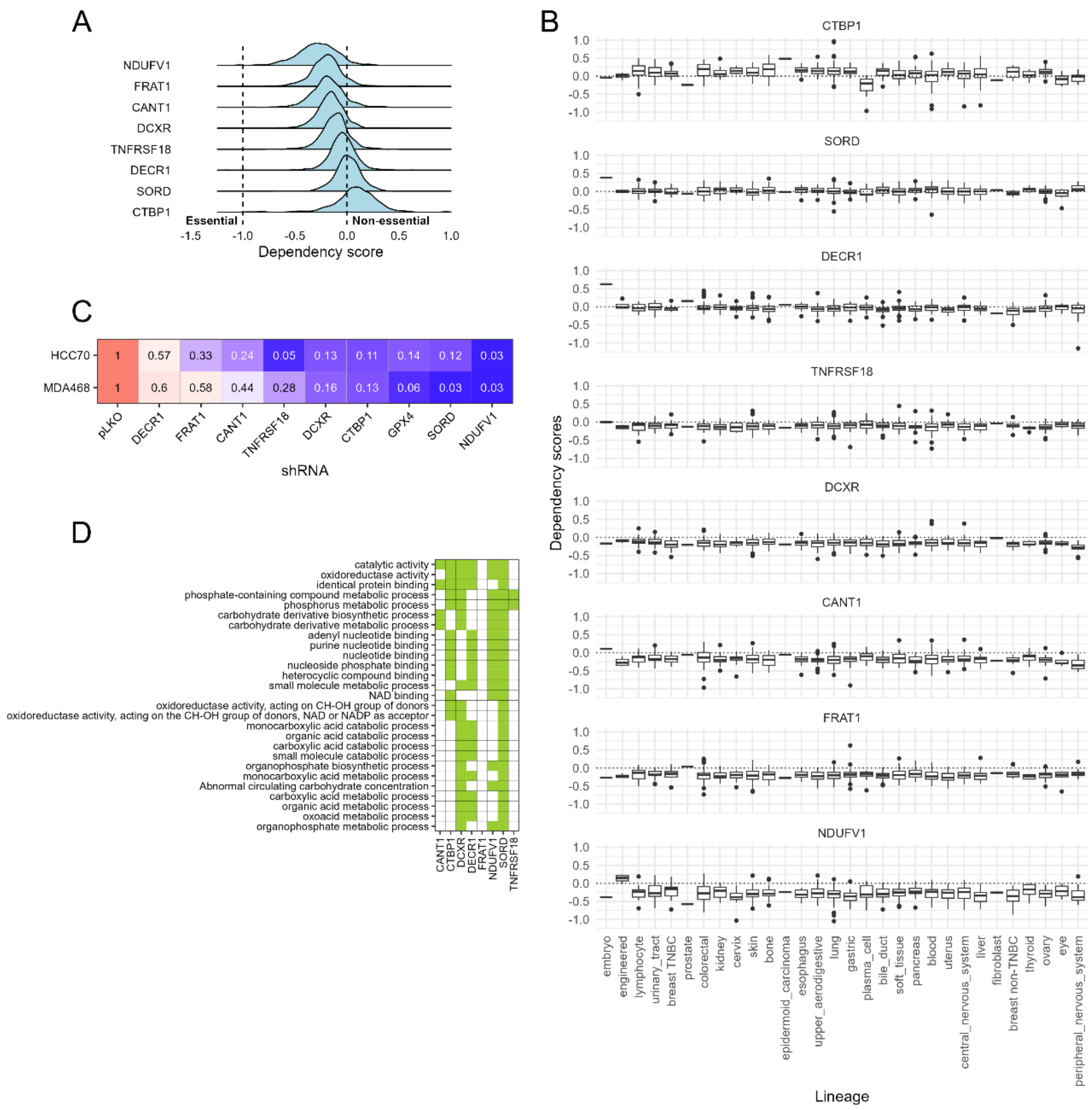

Figure S5. Supporting information for the integration of predictors and validation (related to Figure 6)

(A) Essentiality scores for the genes selected for experimental validation, across all cell lines of all lineages included in the Achilles dataset. **(B)** Essentiality scores for the genes selected for experimental validation, across all cell lines of all lineages included in the Achilles dataset. Boxplot depicts the essentiality scores per lineage. **(C)** Knock-down of the indicated genes was performed in MDA-MB-468 and HCC70 cells using shRNA. qRT-PCR analysis was performed to validate the knock-down efficiency of the shRNA. Numbers indicate fold change in the expression of the gene being knocked-down. **(D)** Gene ontology (molecular functions) was performed for the 8 genes used for validation. Only significantly enriched terms are shown.

Table S3 - List of public ferroptosis inducers datasets (related to Figure 3)

Symbol	Inducer	Model	Dataset reference (GEO)	Paper reference (Pubmed)
F1	Erastin	HT1080	Taken from paper	PMID: 24844246
F2	Erastin	HepG2	GSE104462	PMID: 31108460
F3	Erastin	RCC4	GSE121689	PMID: 31484063
F4	Withaferin A	NB	GSE112384	PMID: 29939160
F5	Erastin	MEF	GSE131444	PMID: 33782392
F6	Ferrotocide	HT29	GSE126868	PMID: 31086302
F7	NaIO3	Arpe-19	GSE142591	PMID: 33895485
F8	ML210	H-STS	GSE96760	PMID: 29915428
F9	Erastin	H-STS	GSE96760	PMID: 29915428
F10	SLC7A11 KO	In-vivo	GSE119628	PMID: 32241947
F11	shGPX4	BeWo	GSE147625	NA
F12	GPX4 KO	Mice Treg	GSE160338	PMID: 34133924
F13	RSL3	MM1	GSE182638	PMID: 34830117
F14	Hemin	OPC (mice)	GSE197104	NA
F15	sevoflurane	U251	GSE193295	PMID: 35372041
F16	ML162	MDA231	GSE162069	PMID: 33854057
F17	NC06	J774M	GSE163399	PMID: 33547170
F18	Erastin	HCC38	GSE154425	PMID: 34040090
F19	NASH liver	in-vivo	Taken from paper	PMID: 32145145

Table S6 – List of primers used in this study (related to Figure 6)

Gene	Forward primer	Reverse primer
DECR1	CATGAAGCTACCGGCCAGGG	AGGCCCAGCTTCCTCTCACA
FRAT1	TGAACCCCACGCCCTGTCTA	CTCTGTCCCGCAACCCAGTG
CANT1	TCGGCCACCTTCCTCCGTC	CAGGCTGGTGCTCTGTGGTC
DCXR	ACCCCCACAAGGCCAAGACTA	TCAGCAGAAAGAGGATGGCGT
CTBP1	TCCACGCAGGAGATCCATGA	CTGCCAATCCGGACGATGAT
GPX4	ACCGAAGTAAACTACACTCAG	GGCGAACTCTTTGATCTCTT
SORD	GCCGGAAGTCACCATCGAGT	AGCACGAGGTTCCCACCAGA
TNFRSF18	CAGGGGGTACAGTCCCAGG	GAGAAACCCGAACTGGGTGC
NDUFV1	AAGGTGACAGCGTGAGGTGAC	TCCGGTCTTCATCCTTCAGCG
Actin	GGGCATGGGTCAGAAGGATT	TCGATGGGGTACTTCAGGGT
